# Outermost Cationic Surface Charge of Layer‐by‐Layer Films Prevents Endothelial Cells Migration for Cell Compartmentalization in Three‐Dimensional Tissues

**DOI:** 10.1002/advs.202417538

**Published:** 2025-02-22

**Authors:** Jinfeng Zeng, Sven Heilig, Matthias Ryma, Jürgen Groll, Congju Li, Michiya Matsusaki

**Affiliations:** ^1^ College of Textiles Donghua University Shanghai 201620 China; ^2^ Department of Applied Chemistry Graduate School of Engineering Osaka University 2‐1 Yamadaoka Suita Osaka 565–0871 Japan; ^3^ University of Würzburg Pleicherwall 2 97070 Würzburg Germany; ^4^ Joint Research Laboratory (TOPPAN) for Advanced Cell Regulatory Chemistry Osaka University Suita Osaka Japan

**Keywords:** basement membrane, cell migration, ECs sprouts, patterned vascular tissue, positively charged nanofilms

## Abstract

Tissues and organs possess an organized cellular arrangement that enables their unique functions. However, conventional three‐dimensional (3D) encapsulation techniques fail to recapitulate this complexity due to the cell migration during cell culture. In biological tissues, basement membranes (BMs) are essential to mechanically support cellular organization. This study finds that a positively charged outermost surface of multilayered nanofilms, fabricated through layer‐by‐layer assembly of poly‐l‐lysine (PLL) and dextran (Dex) via hydrogen bonds, stimulates the barrier functions of BMs. This type of artificial BM (A‐BM) demonstrates enhanced barrier properties in comparison to other types of A‐BMs composed of BM components such as collagen type IV and laminin. Such an enhancement is potentially associated with the outermost cationic layer, which inhibits the sprouting of endothelial cells (ECs) and effectively prevents EC migration over a 14‐d period, aligning with the formation timeline of natural BMs in 3D tissues. Finally, 3D organized vascular channels are successfully engineered with the support of shape‐adaptable PLL/Dex nanofilms. This approach offers a guideline for engineering organized 3D tissue models by regulating cell migration, which can provide reliable platforms for in vitro permeability assay of new drugs or drug delivery carriers.

## Graphical abstract

A nanometer‐sized artificial basement membrane (A‐BM) is developed to replicate the barrier functions of the basement membrane and prevent cell migration. It is demonstrated that the outermost cationic surface charge of nanofilms can effectively inhibit endothelial cell migration and sprouting, allowing for the construction of 3D patterned vascular tissues with the barrier effects of a shape‐adaptable A‐BM.

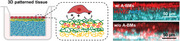

## 1 Introduction

Numerous mammalian tissues possess distinct cellular configurations, characterized by structural patterning and compositional heterogeneity, thereby enabling their unique functions within biological systems.^[^ ^1^ ^]^ However, conventional tissue engineering techniques usually fail to recapitulate this complexity due to the random encapsulation of multiple types of cells. Therefore, to accurately mimic the functionality of living systems, it is essential to develop innovative strategies that ensure reproducibility in recreating the complexity of structures and scalability for widespread application.

Multicompartment models and constructs try to replicate native tissue heterogeneity. Since 1994, there has been a notable recognition of, and endeavor towards, the fabrication of patterns. Ingber et al. designed for the first time a patterned two‐dimensional (2D) substrate using a soft‐lithographic technique to position cells in predetermined locations.^[^ ^2^ ^]^ Advanced methodologies, such as dip‐pen nanolithography,^[^ ^3^ ^]^ nanoimprint lithography,^[^ ^4^ ^]^ and molecular assembly patterning,^[^ ^5^ ^]^ have since been developed for depositing biomolecules individually on 2D substrates, facilitating the evaluation of cell behaviors in 2D patterned co‐culture systems. Further clarification is needed to fully understand how these findings translate to more physiologically relevant three‐dimensional (3D) environments. Primo and Mata reviewed recent techniques for creating 3D patterns of functional molecules within hydrogels.^[^ ^1^ ^]^ Most of these approaches, including 3D photo‐patterning, advanced microfluidics, electric and magnetic fields, as well as precise chemical design, have primarily focused on producing functional biological scaffolds rather than 3D tissues. Zhang et al.^[^ ^6^ ^]^ demonstrated an acoustofluidic system capable of arranging cells within hydrogels, highlighting its potential for constructing biomimetic tissues, such as muscle fibers. However, the selective 3D encapsulation of multiple cell types remains underexplored. To address this, anisotropic spherical hydrogel microparticles composed of distinct extracellular matrix (ECM) hemispheres have been developed to facilitate the co‐culture of multiple cell types. For example, a gas‐shearing strategy was employed to produce microparticles with two, four, six, and even eight compartments, enabling the successful encapsulation of HepG2 and HeLa cells in separate compartments.^[^ ^7^ ^]^ Recently, Hu^[^ ^8^ ^]^ also reported on Janus alginate/poly‐l‐lysine/alginate (APA) microcapsules, which facilitate the spatial organization of multi‐cellular co‐cultures, through a programmable electrodeposition method. Meanwhile, 3D bioprinting techniques have advanced the deposition of multiple cell types and materials. Li et al.^[^ ^9^ ^]^ recently fabricated a liver lobule‐like structure by printing human adipose‐derived mesenchymal stromal/stem cells (hASC) alongside hexagonally patterned human umbilical vein endothelial cells (HUVEC) at the construct's center. The co‐culture of hASC and HUVEC promoted hepatic differentiation and vascularization both in vitro and in vivo. These advancements demonstrate significant progress in the construction of 3D compartmentalized cells and tissues. However, precise cell positioning can be disrupted during the cell culture period due to the cell migration, such as the endothelial cells (ECs) sprouting toward other tissues.^[^ ^10^, ^11^ ^]^ Introducing barriers at the cellular level to inhibit cell migration could be beneficial for maintaining long‐term compartmentalization in multicellular co‐cultures and ensuring orderly tissue differentiation. To date, as far as we know, only the work by Takayama's group^[^ ^12^ ^]^ has succeeded at seeding different types of cells on an existing cell monolayer based on an optimized polymeric aqueous biphasic system (ATPS) of polyethylene glycol and dextran solution. However, cell behaviors in high concentration ATPS solution and the stability necessary to maintain cell compartmentalization are still unclear.

We were inspired by the hierarchical structures of native tissues/organs, where the basement membrane (BM) acts as a support platform to regulate cellular behaviors and adjust the passage of both cells and large molecules.^[^ ^13^, ^14^, ^15^, ^16^ ^]^ The successful construction of artificial BMs (A‐BMs) in vitro might greatly contribute to the fabrication of 3D organized tissue structures. BM has been identified as a dense, continuous sheet‐like structure with nanometer‐scale thickness.^[^ ^17^, ^18^ ^]^ Different strategies have been developed to duplicate the structure and function of BMs, including porous polymer membranes,^[^ ^19^, ^20^ ^]^ native ECM membranes,^[^ ^21^ ^]^ and electrospun scaffolds.^[^ ^22^, ^23^ ^]^ A compartmentalized co‐culture of HUVEC and human astrocytes on either side of a porous PDLLA membrane was successfully established to model the blood‐brain barrier.^[^ ^20^ ^]^ Davalos further introduced BM mimics using ultrathin (≈3 µm thick), ultraporous fibrous networks, which closely resemble physiologically relevant thicknesses, enabling effective crosstalk and physical interaction between cells on either sides.^[^ ^24^ ^]^ Electrospun nanofiber meshes permitted bipolar cultivation of epithelial (endothelial) cells and mesenchymal cells, forming human primary alveolar‐capillary barrier models^[^ ^22^, ^25^ ^]^ and skin models.^[^ ^20^ ^]^ However, applying these techniques to prepare in situ A‐BMs with nanometer‐scale thickness on existing cell layers is challenging, as they require specific apparatus, involve complex fabrication processes, and necessitate the use of organic solvents. Moreover, considering the diversities in structure and shape of tissues among different body parts, such as the tubular configuration in blood vessels, and the irregular lamellar architecture in the liver and intestinal wall, there is a compelling need for advanced methodologies to in situ fabricate adaptable A‐BMs tailored to the variable and intricate architectures within 3D tissues.

**Scheme 1:**
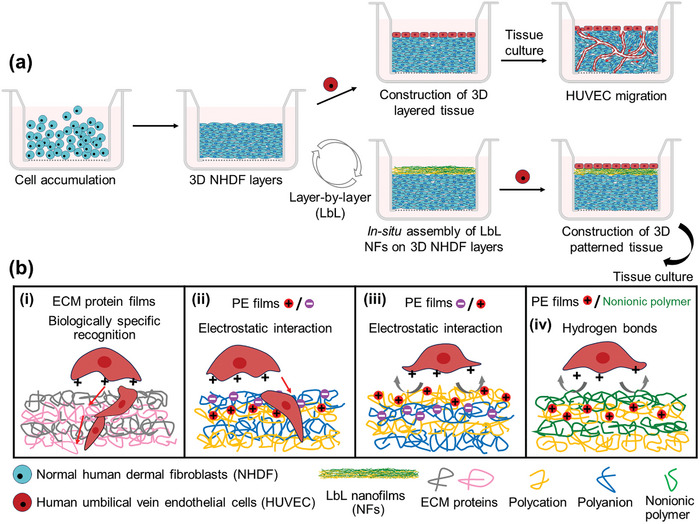
(a) Schematic representation of the development of A‐BMs by *in‐situ* LbL assembly technology on fibroblast tissues to construct 3D organized biological tissues composed of NHDF and HUVEC. In the absence of A‐BMs, HUVEC migrate during tissue culture, thereby altering the 3D hierarchical tissue structure. (b) Comparison of the barrier function of various types of LbL films assembled from different ECM proteins or polymers to inhibit HUVEC migration during tissue culture.

To construct ultrathin nanofilms, the layer‐by‐layer (LbL) assembly technique offers a robust and versatile method for controllable biocoating at a micro‐/nanometer scale. The assembly of multilayered films is achieved by immersing the substrate into different solutions for the alternate deposition.^[^ ^26^, ^27^ ^]^ This straightforward method endows LbL assembly with significant advantages, including the ability to prepare ultrathin films whose thickness is controllable and whose shapes are customized based on the substrate. In our group, a multifunctional A‐BM was fabricated by the assembly of collagen type IV (Col‐IV) and laminin (LM), which are derived from the main components of BMs.^[^ ^28^, ^29^ ^]^ Assembled Col‐IV/LM nanofilms exhibited controllable thickness and size‐dependent molecular permeability. They facilitated cell adhesion, differentiation and enabled effective heterogeneous cellular communication through the porous fiber networks. Moreover, with the assistance of A‐BMs, a patterned cell co‐culture could be maintained for up to 5 d. Without them, ECs migrated and sprouted into fibroblast layers, forming random capillaries (**Scheme** **1**a). This suggests the importance of adaptable A‐BMs in preserving the integrity of compartmentalized cell co‐culture systems. However, it has been reported that a partly plugged mesh of BM proteins were deposited by immortalized alveolar type II epithelial cells cultured with Matrigel in vitro for 5 d and the formation of a thin BM sheet took around 10 d.^[^ ^30^ ^]^ The barrier effect of A‐BMs should, therefore, be further enhanced to keep long‐term cell compartmentalization until the reconstruction of natural BMs in 3D tissues.

It has been reported that directional ECs sprouts tend to form toward the negatively charged membrane surface of apoptotic cells and sprouting ECs typically migrate toward the cathode in a direct current electric field.^[^ ^31^, ^32^, ^33^ ^]^ Moreover, in contrast to the unchangeable conformation of proteins, polyelectrolyte assembled films generally have homogeneous charge distribution, adaptable conformation, and are more stable when co‐cultured with cells. This suggests that a polyelectrolyte nanofilm with a positively charged outermost layer could effectively prevent the migration of ECs. For proof of concept, we constructed a series of ECM protein films and polyelectrolyte films to mimic the barrier functions of BMs (Scheme 1a). The barrier properties of the LbL nanofilms were greatly influenced by their outermost surface charge, where the effective nanofilms have a cationic surface while the surfaces of the ineffective ones are negatively charged (Scheme 1b). As a case study, we evaluated the cell adhesion properties and barrier functions of a cationic nanofilm, created through hydrogen bonds between poly‐l‐lysine (PLL) and dextran (Dex), in comparison to previously reported Col‐IV/LM NFs. A marked enhancement in the barrier properties was found to effectively inhibited cell migration throughout the 14‐d experimental period. To highlight the LbL assembly technique's capability for fabricating customizable A‐BMs in diverse tissue forms, 3D organized tubular vascular channels were successfully engineered by in situ PLL/Dex NFs formation on smooth muscle cell (SMC) layers, followed by establishing a confluent EC monolayer. This study provides a guideline for engineering organized 3D tissue models by regulating cell migration. Such models could yield more dependable outcomes for new drug screening methods and nanotoxicology assessments.

## 2 Results and Discussion

### 2.1 Fabrication of PLL/Dex NFs

**Figure 1:**
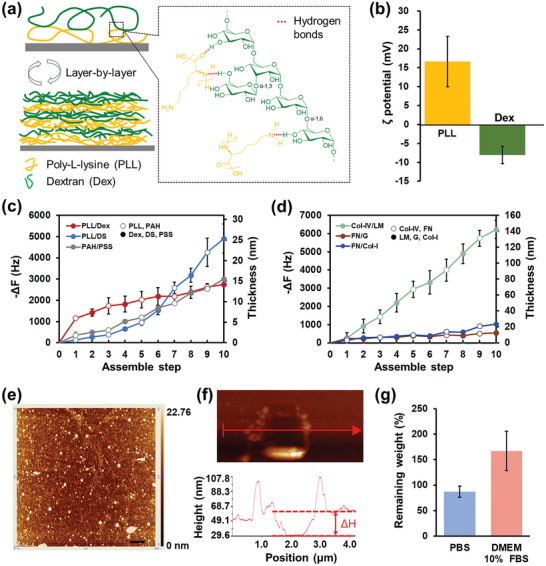
Buildup of PLL/Dex multilayered ultrathin nanofilms (NFs). (a) Schematic illustration of the LbL assembly process of PLL and Dex via hydrogen bonds. (b) ζ potential of PLL (0.1 wt%) and Dex (0.1 wt%)/Tris‐HCl buffer solutions (50 mM, pH = 7.4, 37 °C), n = 3. (c) & (d) Frequency shifts and film thickness increases during assembly are summarized for various LbL films composed of polymers or ECM proteins. Frequency changes were recorded by QCM during assembly in 50 mM Tris‐HCl buffer solution (pH = 7.4, 37 °C). The concentration of each polymer or protein is 0.1 wt%. (e) AFM tapping mode image (10 × 10 µm) of multilayers composed of PLL and Dex. Scale bar: 1 µm. (f) Scratching with the AFM tip creates a square hole of the PLL/Dex NFs, where the depth ΔH corresponds to the film thickness. (g) The remaining weight percentage of PLL/Dex NFs after immersion in PBS and DMEM/10% FBS for 60 min at 37 °C, which is summarized from the frequency change recording by QCM.

The LbL assembly technique has been demonstrated to be a highly effective method for the in situ fabrication of shape‐customized nanofilms in 3D tissues, which hold great potential to serve as A‐BMs to facilitate compartmentalized co‐culture of cells.^[^ ^28^, ^29^ ^]^ In the present study, PLL and Dex were used as foundational components for the construction of cationic films, as illustrated in **Figure** **1**a. PLL is a well‐known polycation, comprising a polypeptide derived from the essential amino acid L‐lysine. At physiological pH, each repeating unit of PLL carries a positive charge on the amine, whose ζ‐potential was measured at 16.7 ± 6.7 mV (Figure 1b). Dex is characterized as a nontoxic, hydrophilic polysaccharide, consisting of d‐glucopyranose repeating units linked through glycosidic linkages. Although it is known that Dex has zero net charge, an abundance of uniformly distributed hydroxyl groups in Dex form intermolecular hydrogen bonds with amine and amide groups of PLL.^[^ ^34^ ^]^ PLL/Dex multilayer nanofilms were fabricated for the first time by alternating deposition of the two polymers through the hydrogen bonds between PLL and Dex (Figure 1a), resulting in a net positive surface charge. This assembly process was assessed using a quartz crystal microbalance (QCM). As shown in Figure S1 (left) (Supporting Information), a decrease in frequency was observed with each assembly step, suggesting the successful alternate deposition of PLL and Dex. Throughout the assembly process, the variation in film thickness was determined based on the changes in frequency according to Sauerbrey's equation,^[^ ^35^ ^]^ as recorded by QCM (Figure 1c). Upon reaching the assembly of 5 bilayers, the thickness of the deposited film was ≈14.2 ± 0.47 nm. In the same way, the electrostatic interaction‐driven LbL films comprising PLL and dextran sulfate (PLL/DS), poly(allylamine hydrochloride)/poly(styrene sulfonate) (PAH/PSS) showed clear step‐by‐step increases in −Δ*f*. For performance comparison with PLL/Dex films, Col‐IV/LM, FN/G, and FN/Col‐I films were also developed via the specific biological interaction (Figure 1d). Furthermore, the morphological characteristics of PLL/Dex NFs were analyzed using atomic force microscopy (AFM), as depicted in Figure 1e. The AFM image clearly demonstrated the development of a smooth and uniform film with a roughness of 4.08 ± 0.85 nm. Hundreds of scratch tests were conducted using an AFM tip in contact mode on a small area (1 × 1 µm) to measure the thickness of the deposited films. The thickness of PLL/Dex NFs, defined by the height difference between the scratched and unscratched areas, was ≈20 nm (Figure 1f). The lower value of thickness estimated by QCM from Sauerbrey's equation may be due to the fact that it does not take into account the viscoelasticity of the soft gel‐like film in the swollen state, which is usually formed by weak interactions.^[^ ^36^ ^]^ Moreover, the correlation between the increase in film thickness and the number of assembly steps, as quantified by QCM, illustrates that the film thickness, measured on the nanometer scale, can be easily adjusted. This capability highlights the precision and flexibility of the LbL assembly technique in tailoring nanofilm dimensions to specific requirements.

The nanofilm stability in the physiological environment was also evaluated using QCM at 37 °C. As shown in Figure S1 (right) (Supporting Information), upon immersion in PBS, the frequency of assembled PLL/Dex NFs (blue line) initially exhibited a slight increase because of the removal of unabsorbed polymers, as well as the partial disassociation of assembled nanofilms. It then remained stable and the remaining weight percentage of nanofilm was around 87% after immersion for 60 min, indicating the stability of assembled PLL/Dex NFs (Figure 1g). Conversely, the remaining weight percentage of the nanofilm immersed in Dulbecco's modified eagle medium (DMEM)/10% fetal bovine serum (FBS) exceeded 160% (indicated by the red line), a phenomenon attributable to the gradual attachment of proteins on the film surface. In conclusion, the ability of PLL/Dex NFs to precisely control thickness at the nanoscale combined with their stability in physiological conditions establishes a robust basis for their application as A‐BMs in the in vitro construction of 3D tissue structures.

### 2.2 Cell Adhesion on PLL/Dex NFs

Since both endothelial and mesenchymal cells are co‐localized nearby and adhere well to the natural BMs, cell adhesion on the fabricated A‐BMs were systematically evaluated. The cell adhesion process involves the interaction between integrins in cell membrane and adhesive proteins on the substrate surface, such as fibronectin (FN). The protein adsorption on the films was therefore investigated, as shown in Figure S2 (Supporting Information), and quantified by QCM. Compared to the Col‐IV/LM NFs that demonstrated favorable A‐BMs performance and function as reported in our previous study,^[^ ^28^, ^29^ ^]^ the absorption of both bovine serum albumin (BSA) and fibronectin (FN) on PLL/Dex NFs was higher due to their positively charged surface, which promotes the subsequent cell adhesion.^[^ ^37^ ^]^ The observed low protein adsorption rate on Col‐IV/LM NF may be attributed to the synergistic effects of film disassembly, which is influenced by the attraction of proteins in solution, and the subsequent attachment of these proteins. Figures S3 and S4 (Supporting Information) depict the cell adhesion and spreading morphology on different films including ECM protein, polyelectrolyte films by cytoskeletal staining. Both fibroblast (normal human dermal fibroblast cells [NHDF]) and endothelial cells (HUVEC) adhered well to the cell culture inserts (w/o NFs) and Col‐IV/LM NFs owing to the inherently cell‐friendly nature of these matrices. Comparable cell spreading results were also observed on PLL/Dex NFs, displaying their characteristic morphologies. In contrast, due to the hydrophilic nature of dextran sulfate, PLL/DS NFs inhibited cell adhesion and spreading, and cells remained heavily aggregated even after 2 d of incubation, especially HUVEC. Quantitative analysis further demonstrated that PLL/Dex NFs exhibited enhanced cell adhesion properties relative to PLL/DS NFs, thanks to their positively charged surfaces. There was no significant difference in cell spreading area and adhered cell number on PLL/Dex NFs compared to the cell‐friendly substrates, highlighting the favorable cell adhesion properties of the prepared PLL/Dex NF.

Furthermore, ECs attach tightly and align to BMs, with their function being regulated by these BMs. The functions of ECs on the developed A‐BMs were, therefore, investigated through 2D culture of HUVEC, including the expression of endothelial marker CD31 and tight junction protein, zonula occludens‐1 (ZO‐1). CD31 is an adherent molecule highly enriched at interendothelial junctions of vascular endothelial cells and is involved in angiogenesis, vascular integrity, and remodeling.^[^ ^38^ ^]^ ZO‐1 is a tight junction protein and plays an important role in the maintenance and regulation of epithelial/endothelial barrier functions.^[^ ^39^ ^]^ Figure S5 (Supporting Information) illustrates that, in addition to PLL/DS NFs, a cobblestone‐like morphology of HUVEC was observed on all substrates, with no marked differences noted. However, HUVEC displayed poor spreading on PLL/DS NFs due to the inhibitory effect of DS on cell attachment. The distribution and margin of both CD31 and ZO‐1 were more distinct on Col‐IV/LM NFs compared to PLL/Dex NFs or the naked insert membrane, because of the role of BM proteins in enhancing differentiation of vascular endothelial cells.^[^ ^40^ ^]^ Meanwhile, the uniform and continuous signals of CD31 and ZO‐1 on the edge of cell–cell joints demonstrated the barrier integrity of an endothelial monolayer culture on PLL/Dex NFs.^[^ ^41^ ^]^ Overall, the findings concerning cell adhesion and functions of ECs highlight the potential of PLL/Dex NFs as a robust scaffold capable of supporting endothelial and mesenchymal cells. Their nanoscale thickness and stability in physiological environments provide further insights into their suitability as A‐BMs in tissue engineering. The subsequent sections will concentrate on the barrier properties of the developed A‐BMs during long‐term tissue culture.

### 2.3 Barrier Function of LbL Films on 3D Patterned Multicellular Co‐Culture

**Figure 2:**
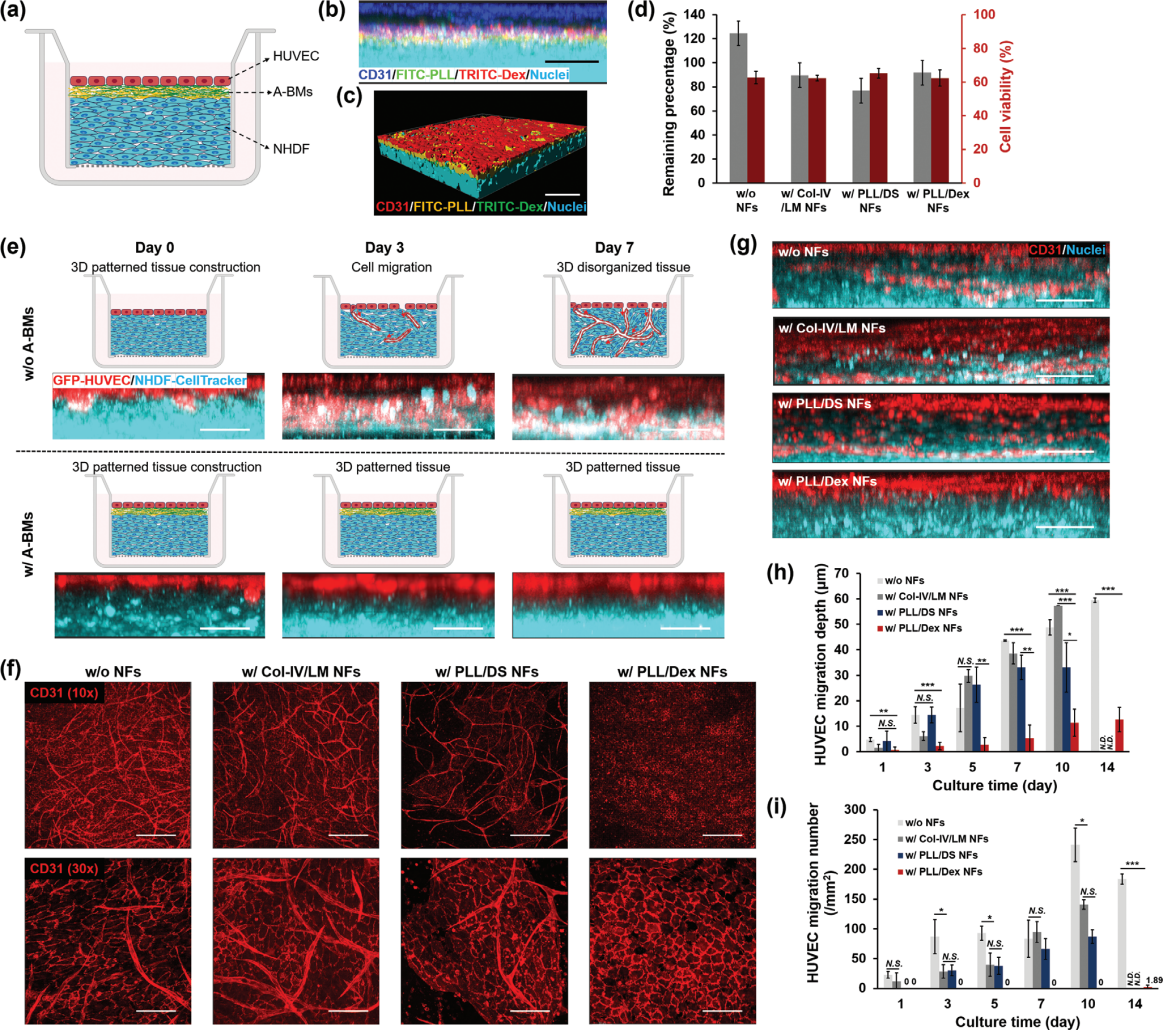
Barrier function of multilayered nanofilms on the patterned co‐culture of NHDF and HUVEC. (a) Schematic representation of the constructed 3D patterned tissue structure composed of HUVEC, A‐BMs and NHDF. (b) Cross‐sectional CLSM image showing the location of PLL/Dex NFs (FITC conjugated PLL in green and TRITC conjugated Dex in red) between the HUVEC monolayer (immunostained for CD31 in blue) and NHDF layers (stained with Hoechst 33 342 in cyan). Scale bar: 50 µm. (c) 3D reconstructed CLSM image of compartmentalized cell co‐culture system. Scale bar: 100 µm. (d) Cell number counting (gray and left) and cell viability (red and right) of NHDF layers after *in‐situ* LbL assembly treatment and then cultured overnight, n = 3. (e) up: Schematic representation of the structure of 3D tissue during 7 days of culture. In the absence of any physical barrier, HUVEC gradually migrate towards the fibroblast tissues, thereby altering their organized structure. With the support of A‐BMs, the 3D organized tissue structure is preserved by inhibiting the migration of HUVECs. bottom: Cross‐sectional CLSM images show the changes in the relative positions of NHDF and HUVEC during the tissue culture. HUVEC are labeled with GFP (shown in red) and NHDF are stained with CellTracker™ Deep Red (shown in cyan). Scale bar: 50 µm. (f) Immunostaining of HUVEC with CD31 visualized by CLSM. The 3D scanning results of the co‐culture system, from top to bottom, are compressed to a single image. up: observed with 10 × magnification, scale bar: 300 µm. bottom: observed with 30 × magnification, scale bar: 100 µm. (g) Cross‐sectional CLSM images of co‐culture system with different LbL films observed after 7 days of culture. HUVEC are stained with CD31 shown in red and all cell nuclei are stained with Hoechst 33 342 in cyan to show the position of NHDF. Scale bar: 50 µm. (h) HUVEC migration depth (compared with the position of HUVEC at Day 0) and (i) number of migrated HUVEC towards NHDF layers during the co‐culture with different LbL films for 14 days. (h) n = 3, (i) n = 6. *p < 0.05, **p < 0.01, ***p < 0.001. *N.S*. no significant difference. *N.D*. no data.

Tissue engineering has garnered considerable interest for its potential in constructing 3D tissues or organs. While precise control over cell localization in a 3D construct is achievable, maintaining the ordered structure of tissues over time in culture presents challenges due to cell migration. In particular, migration of ECs plays a crucial role in angiogenesis, and they readily migrate toward attractors such as basic fibroblast growth factor secreted by NHDF.^[^ ^11^ ^]^ Consequently, to replicate the organized and layered tissue structure, where natural BMs are distributed between endothelial (epithelial) cells and connective tissues in vivo, PLL/Dex NFs were assembled in situ between fibroblast layers and the ECs monolayer (**Figure** **2**a). In this section, the barrier effect of PLL/Dex NFs, which act as A‐BMs to block ECs migration and maintain 3D patterned multicellular co‐cultures, was estimated. We also compared the barrier effect of other candidate A‐BMs, including Col‐IV/LM NFs reported in previous studies^[^ ^28^, ^29^ ^]^ and a polyelectrolyte LbL film composed of PLL and DS.

The developed 3D patterned multicellular structure and the location of PLL/Dex NFs were confirmed by cross‐sectional confocal laser scanning microscopy (CLSM). Assembled fluorescein isothiocyanate (FITC)‐PLL/tetramethylrhodamine (TRITC)‐Dex NFs was observed to locate between the HUVEC monolayer and the NHDF layers, forming a “sandwich” structure (Figure 2b). A 3D‐constructed CLSM image shown in Figure 2c further verified the distribution of nanofilms between different cells. Meanwhile, the long‐term stability of the nanofilms was investigated by quantifying changes in fluorescence intensity (FI) of each polymer during cell co‐culture (Figure S6, Supporting Information). The FI on Day 1 was set as the baseline (100%), and subsequent nanofilm retention was quantified relative to this. Both PLL and Dex underwent enzymatic hydrolysis during incubation with cells. Dex showed a gradual decrease in weight, while PLL showed a faster decline within the first 5 d before stabilizing. After 7 and even 14 d of co‐culture, ≈50% of the polymers remained. Considering the facts that the charge originates only from PLL, while Dex is uncharged, PLL/Dex nanofilms maintained their positive charge despite gradual degradation, effectively inhibiting endothelial cell migration throughout the culture period. To develop the A‐BMs between different cells, the alternate deposition of PLL and Dex was carried out in situ on NHDF layers. The dipping and washing process with 50 × 10^−3^ m of Tris‐HCl buffer solution (pH 7.4) for around 2.5 h was expected to induce the detachment or death of cells due to the absence of glucose. Cell numbers and cell viability were then assessed after 24 h of culture. As expected, cell counts for samples subjected to LbL assembly were approximately 43% lower than those for the untreated control sample, indicating cell loss during the assembly process (Figure 2d). However, there was no difference in cell survival rate with or without dipping assembly, which was ≈87%. The 13% mortality in all groups was caused by 20 min of enzymatic digestion using 0.25% trypsin and 0.02% EDTA during the cell isolation. The biocompatibility of the in situ dipping assembly process for nanofilm fabrication in 3D tissues was further demonstrated by the live/dead staining assay as shown in Figure S7 (Supporting Information). Few dead cells could be observed in each tissue, indicating the mild nature of the assembly process.

During tissue culture, the relative position of HUVEC and NHDF was monitored through CLSM, as shown in Figure 2e. Consistent with previous studies,^[^ ^28^ ^]^ the HUVEC monolayer was initially localized hierarchically on the tissue surface and subsequently migrated into NHDF layers, extending even to the tissue bottom within 3 d in the control group lacking any physical barrier. However, under the barrier effect of A‐BMs between HUVEC and NHDF, the migration of HUVEC was effectively prevented during the 7 d of co‐culture. Moreover, in the human body, the innermost layer of blood vessel lumen consisted of a monolayer of ECs lining the interior surface. These intact ECs attach and align tightly to BMs, forming a squamous confluent layer.^[^ ^42^ ^]^ A CD31‐marked confluent HUVEC monolayer was observed in Figure 2e, indicating the formation of a continuous endothelial layer on PLL/Dex NFs. However, in the control sample without A‐BMs and in a co‐culture system using Col‐IV/LM NFs, not only monolayers of HUVEC were found, but also elongated HUVEC and cross‐capillary networks. These results are related to migration and sprouting of ECs, and these phenomena are undesirable when constructing well‐organized vascular tissues. Migrated HUVEC were also observed in the group containing PLL/DS NFs, despite the poor cell adhesion and an incomplete endothelial layer on PLL/DS NFs. In addition, cross‐sectional CLSM images provided substantial evidence of the barrier effect of PLL/Dex NFs in compartmentalized tissue cultures, effectively restricting HUVEC to the tissue surface as depicted in Figure 2f. In contrast, ECs migration into NHDF layers was clearly found in the other samples. This migration was further elucidated through a video (Video S1, Supporting Information), which depicted the HUVEC movement into NHDF layers within a 3D tissue structure viewed from top to bottom. Histological analyses (Figure S8, Supporting Information) also confirmed these findings that CD31‐marked HUVEC localized on the surface of 3D tissues in the presence of PLL/Dex NFs. But, HUVEC were observed in the middle of 3D tissues, even forming capillary lumen, in the other samples.

Considering that BM proteins would be secreted and deposited during the tissue culture for at least 10 d,^[^ ^30^ ^]^ the long‐term patterned culture was investigated. The relative position of HUVEC, as well as the migrated depth (Figure 2g, relative position changes of HUVEC compared with their position on Day 0) and migrated cell number (Figure 2h), were monitored and quantified over a 14‐d co‐culture period through the analysis of 3D cross‐sectional CLSM images shown in Figure S9 (Supporting Information). Consistent with the analysis in Figure 2e, without any barrier, migrated HUVEC in the control sample were observed on the first day of co‐culture. Despite the evidence that Col‐IV and LM possess specific cell‐binding sites to guide cell adhesion and migration, the inhibitory capacity of Col‐IV/LM NFs was limited to a maximum of 3 d. This limitation is due to the fact that these protein membranes are destabilized during co‐culture and are subject to enzymatic degradation, rendering them incapable of efficiently regulating cell migration in the long term.^[^ ^29^ ^]^ On the contrary, the migration of HUVEC was completely inhibited by PLL/Dex NFs, preserving the compartmentalized tissue structure for approximately two weeks. A comparative analysis revealed that negligible cell migration was observed after a 14‐d culture due to the barrier effect of PLL/Dex NFs, and the minimal migration depth counted was a result of the cell proliferation‐induced changes in tissue thickness. Both the number of migrated HUVEC and the depth of their migration were significantly greater in the group treated with PLL/DS NFs than in those treated with PLL/Dex NFs. This happened despite the fact that PLL/DS NF and PLL/Dex NF were assembled from virtually the same polymers, including polylysine and dextran/derivatives. Moreover, studies have shown that ECs can penetrate dextran methacrylate hydrogels to establish new vascular formations, suggesting that Dex does not inhibit EC migration.^[^ ^43^ ^]^ The mechanism by which PLL/Dex NFs prevent cell migration will be discussed in the following section. In summary, through the research discussed above, superior performance of PLL/Dex NFs in preventing HUVEC migration for up to two weeks was confirmed, indicating their potential to act as an effective barrier of A‐BMs for compartmentalized tissue culture.

### 2.4 Effects of the Outermost Surface Charge on Cell Migration

**Figure 3:**
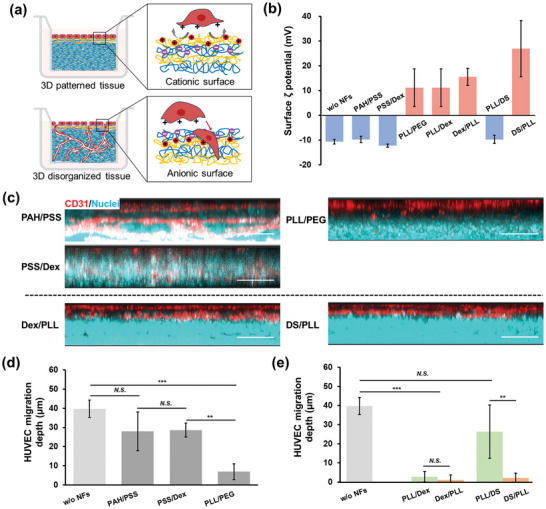
Effects of film outermost surface charge on HUVEC migration. (a) Schematic illustration depicts the correlations between the barrier functions of LbL films in preventing cell migration and preserving the organized tissue structure and their outermost surface charge. (b) Surface ζ potential of various polyelectrolyte multilayered nanofilms assembled on a glass substrate in 50 mM Tris‐HCl buffer solution (pH 7.4) at 37 °C. The measurement of ζ potential is performed in the same buffer solutions suspended with Nile Red‐polystyrene nanoparticles as tracer particles (0.0005 wt%) at room temperature, n = 3. (c) Cross‐sectional CLSM images of a co‐culture system with various LbL films, which are observed after 7 days of culture. Scale bar: 50 µm. (d) and (e) HUVEC migration depth through various LbL NFs after 7 days of co‐culture. n = 3, **p < 0.01, ***p < 0.001. *N.S*. no significant difference.

To investigate why PLL/Dex NFs possess superior performance to both Col‐IV/LM NFs and PLL/DS NFs in preventing HUVEC migration, a series of LbL films were constructed for the patterned cell co‐culture (**Figure** **3**), including polyelectrolyte and ECM protein multilayered films, as well as Matrigel. The LbL films’ assembly process was recorded by QCM (Figure 2d and Figure S10, Supporting Information). The cross‐sectional CLSM images in Figure S11 (Supporting Information) show the relative position of GFP‐HUVEC (in green) and NHDF (stained with CellTracker Deep Red) in the presence of ECM protein nanofilms at Day 7. However, these nanofilms, including Col‐IV/LM, FN/Col‐I, and FN/G, failed to prevent the migration of HUVEC. Only the patterned co‐culture structure was maintained with a Matrigel coating. Matrigel, derived from Engelbreth–Holm–Swarm tumor BMs, consists of almost the same components as natural BMs but raises safety concerns due to its tumorigenic origin.^[^ ^44^ ^]^ Further analysis (Figure 3c,d) compared the effectiveness of LbL films formed via electrostatic interactions versus those formed via hydrogen bonds. Interestingly, films formed from polycationic and nonionic polymers played a better role in preserving the patterned tissue structure than their electrostatically assembled counterparts. This difference in performance appears to be related to the surface ζ‐potential of the films, with the effective nanofilms have a positively charged surface while the surfaces of the ineffective ones are negatively charged (Figure 3b).

To further investigate the effects of the outermost surface charge on cell migration, nanofilms starting with DS and ending with PLL (DS/PLL NFs) were prepared, which possessed as a positively outermost surface charge, and found to be effective in preventing HUVEC migration, as shown in Figure 3c (bottom) and 3e. Conversely, PLL/DS NFs with negatively charged surfaces were ineffective in blocking HUVEC migration, as discussed above. In addition, negligible cell migration was found in the presence of both PLL/Dex and Dex/PLL NFs, both of which have positively charged surfaces. The aforementioned data indicate that the composition of these films is not the primary factor inhibiting cell migration and sprouting, rather, the surface charge plays a critical role. This experimental phenomenon can be explained by the findings of Federer's group. They found that apoptotic cells induce ECs sprouting through the phenotypic expression of a negatively charged membrane surface.^[^ ^31^ ^]^ They proposed a concept, based on their observations, that sprouting cells exhibit a strongly positively charged surface, as evidenced by the binding of anionized ferritin to sprouting ECs. While the exact source and nature of this positive surface charge on ECs during sprouting remain unclear, these observations are consistent with reports that ECs tend to elongate and migrate toward the cathode in a direct current electric field.^[^ ^32^, ^33^ ^]^ Their hypothesis is also supported well by our findings that the outermost cationic surface charge of nanofilms inhibits the migration or elongation process of HUVEC, despite the fact that HUVEC itself tends to sprout in response to basic fibroblast growth factor secreted by the underlying NHDF.^[^ ^45^ ^]^ These positively charged nanofilms permitted compartmentalized co‐culture of fibroblasts and ECs, acting like a barrier, suggesting that they have the potential to serve as A‐BMs for the construction of highly organized 3D vascular tissues.

### 2.5 Formation of Natural BMs

**Figure 4:**
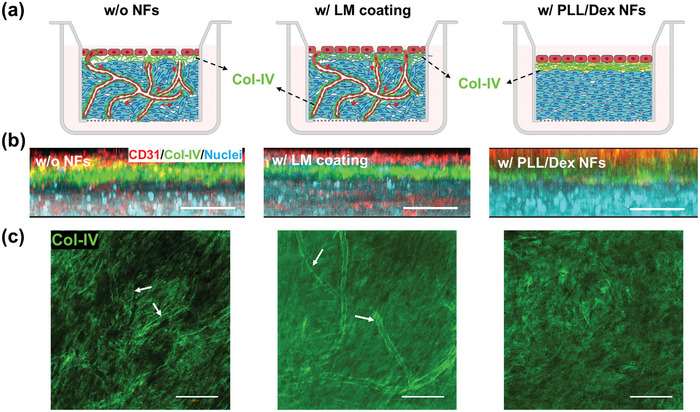
Formation of natural BMs in 3D tissues. (a) Schematic illustration of the assembly and position of Col‐IV that is the main component of natural BMs between different cells in the co‐culture system. (b) Relative position of Col‐IV between HUVEC monolayer and NHDF layers after 7 days of culture. HUVEC is marked by CD31 in red, Col‐IV is immunofluorescence stained in green, and nuclei are stained with Hoechst 33 342 in cyan to show the location of NHDF. Scale bar: 50 µm. (c) Immunofluorescence staining of Col‐IV in 3D tissues. White arrows indicate the assembly of secreted Col‐IV along the capillary wall, where the capillary network is formed due to the migration of HUVEC into the fibroblast tissue. Scale bar: 300 µm.

Natural BMs are supposed to deposit between different cells to fulfill their role in supporting and maintaining the integrity and organization of tissue structures, while ensuring the compartmentalized multicellular co‐culture and enabling engineered tissues to mimic the complex functions of native tissues.^[^ ^30^ ^]^ Col‐IV and LM are identified as the primary components of BMs, and are secreted during tissue culture. Therefore, the deposition of Col‐IV was evaluated over 7 d in 3D tissues (**Figure** **4**a). To avoid the interference of exogenous Col‐IV, a coating of LM was applied instead of using Col‐IV/LM NFs. Deposited Col‐IV was observed to be mainly distributed between the HUVEC monolayer and NHDF layers (Figure 4b), forming dense fibrous networks. However, beneath the monolayer of HUVEC, the fluorescence signals of Col‐IV were also detected in the samples without A‐BMs or with LM coating, especially around the capillary lumen structure, as shown around the white arrows in Figure 4c. Moreover, the LM coating generally enhances cell adhesion and growth, subsequently facilitating the secretion of Col‐IV. The fluorescence intensity of Col‐IV in the sample consisting of PLL/Dex NFs was slightly lower, but still comparable to the sample with LM coating (Figure S12, Supporting Information). There was no significant difference among the three samples. Taken together, these findings confirm the deposition of Col‐IV in the presence of the PLL/Dex NFs barrier, suggesting the formation of natural BM in the long‐term, organized tissue culture.

### 2.6 3D Patterned Vascular Structure

**Figure 5:**
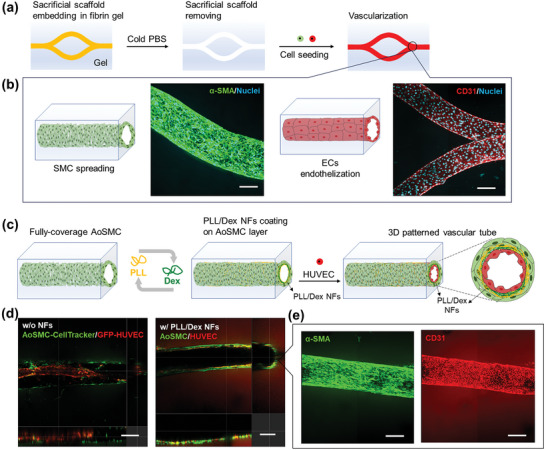
Fabrication of 3D patterned vascular structure. (a) Schematic illustration of cells seeded within microchannels in fibrin gels that were formed by sacrificial scaffold fixation and scaffold dissolution washing with cold PBS. (b) Schematic and CLSM images of the α‐SMA positive AoSMC layer and CD31 positive HUVEC monolayer in microchannels. Nuclei are stained with Hoechst 33 342. Scale bar: 200 µm. (c) Schematic representation of the *in‐situ* LbL assembly of PLL/Dex NFs on AoSMC layers in the microchannels, and the construction of a 3D patterned vascular tube composed of AoSMC layers and a HUVEC monolayer, facilitated by the support of PLL/Dex NFs between the cells. (d) Orthogonal views (xy, xz, and yz) of the CLSM images illustrate the colocalization of AoSMC and HUVEC after culture for 5 days. Left: without PLL/Dex NFs, GFP‐HUVEC detach from the AoSMC layer (labeled with CellTracker™ Deep Red) and aggregate, blocking the microchannel. Right: with the assistance of PLL/Dex NFs, confluent HUVEC monolayer (marked with CD31) is surrounded by compartmentalized AoSMC (stained with α‐SMA). Scale bar: 200 µm. (e) Top‐view of CLSM images demonstrate the morphology of the spreading AoSMC layers and the confluent HUVEC monolayer in the 3D patterned vascular tube. Scale bar: 200 µm.

Biological systems display inherent hierarchical structures, as illustrated by the specialized tubular architecture of blood vessels, comprising ECs, SMCs, and adjacent connective tissues. In the vasculature, BMs typically provide foundational support to the endothelial lining and are closely associated with SMCs.^[^ ^46^ ^]^ The precise mimicking of these structures is crucial for the wide applications of engineered tissue models. In this section, a 3D patterned vascular structure was engineered through the sequential seeding of SMCs and ECs within tubular microchannels, constructed using sacrificial templating methods. PLL/Dex NFs were in situ assembled between ECs and SMCs, functioning as A‐BMs to preserve the organized architecture. As shown in **Figures** **5**a and S13 (Supporting Information), thermoresponsive poly(2‐cyclopropyl‐2‐oxazoline) (P*cyclo*PrOx) scaffolds, fabricated via melt electrowriting (MEW) as detailed in a previous study,^[^ ^47^ ^]^ were embedded in fibrin gel stabilized in a bioreactor. These scaffolds were then dissolved in cold PBS, resulting in the formation of interconnected microchannels, measuring 10 mm in length and 500 µm in diameter (Figure S14, Supporting Information). As shown in Figure 5b and Figure S15a (Supporting Information), the full extension and coverage of human aortic smooth muscle cells (AoSMC) with a spindle‐shaped morphology was confirmed, and a confluent endothelial monolayer was also found on the inner side of the microchannel. Following the alignment of AoSMC, LbL assembly of PLL and Dex was carried out in the circular microchannel to form A‐BMs on the AoSMC layer, followed by the seeding of HUVEC on these films (Figure 5c). The CLSM image in Figure 5d (right) displays a clear tubular double‐layered vascular structure in the presence of PLL/Dex NFs, which was composed of an outer AoSMC layer and inner confluent HUVEC layer (Figure 5e and Figure S16, Supporting Information). Video S2 (Supporting Information) also clearly shows the 3D patterned circular vascular structure. Although no migration of HUVEC was observed during the tissue culture, this may be due to the fact that the AoSMC attached in microchannels were almost monolayer. As shown in Figure S17 (Supporting Information), during layered co‐culture of SMCs and ECs performed in a 24‐well insert, the cross‐sectional CLSM images demonstrate that in the absence of PLL/Dex NFs, migration, and sprouts of HUVEC were observed, while the patterned structure of HUVEC and AoSMC was maintained with the assistance of A‐BMs. Furthermore, without the support of PLL/Dex NFs, the HUVEC monolayer in direct contact with the underlying AoSMC was unstable and prone to detachment from the AoSMC layer, forming aggregates that led to microchannel blockage (Figure 5d (left) and Figure S15b, Supporting Information). This observation suggests the critical role of PLL/Dex NFs, serving as A‐BMs, in providing a supportive platform essential for preserving the integrity and stability of the vascular structure.

### 2.7 Permeability Assay of 3D Patterned Vascular Structure

**Figure 6:**
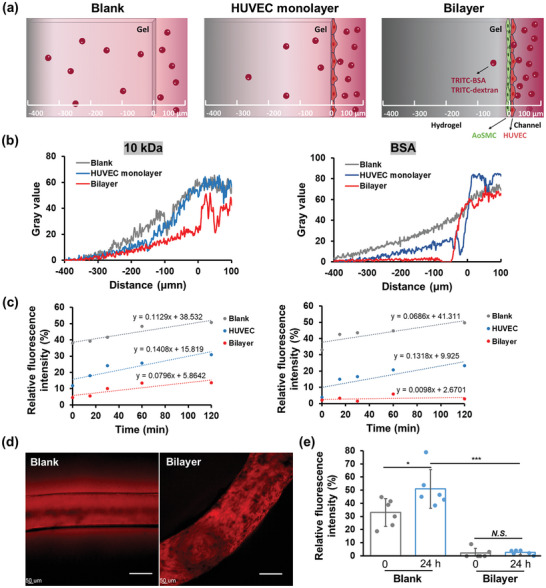
Permeability assay of the 3D patterned vascular structure. (a) Schematic illustration depicting the penetration of 10 kDa dextran and BSA from microchannel (right) into hydrogels (left) under three conditions: without cells (blank), with a HUVEC monolayer or with a bilayer structure. Dextran‐10 kDa and BSA were labeled with TRITC, whose penetration were evaluated by the analysis of fluorescence intensity in hydrogels. (b) Line‐scanning profile showing the fluorescence intensity variation as a function of distance from the microchannels, corresponding to the data in (a), measured at 2 h. (c) Relative fluorescence intensity at a distance of 50 µm from the hydrogel interface, compared to the value at the center of the microchannels, measured at 0, 15, 30, 60, and 120 min. (d) Fluorescence images at 2 h after perfusion of TRITC‐BSA in microchannels. Left panel: without cells in the microchannel; right panel: bilayer of AoSMC and HUVEC in the microchannel. Scale bar is 200 µm. (e) Relative fluorescence intensity of TRITC‐BSA at a distance of 50 µm from the hydrogel interface, measured at 0 and 24 h. *N.S*. no significant difference. *p < 0.05, ***p < 0.001.

Endothelium plays a critical role in controlling the exchange of fluids and solutes, such as plasma proteins and cells, between the bloodstream and surrounding tissues.^[^ ^48^ ^]^ Under physiological conditions, most microvessels exhibit limited permeability due to the endothelial barrier, which restricts the passage of macromolecules and cells. This barrier consists of a continuous endothelium supported by BMs and some pericytes.^[^ ^49^, ^50^ ^]^ BMs and surrounding tissues, such as smooth muscle cells, are essential for maintaining the integrity and stability of the vascular endothelium.^[^ ^51^, ^52^ ^]^ To evaluate the barrier function of the constructed 3D patterned microvascular structure, we examined the diffusion of fluorescently labeled dextran and bovine serum albumin (BSA) from the microchannels into the surrounding hydrogel matrix (**Figure** **6**a). The extent of molecular diffusion was quantified by measuring the fluorescence intensity of fluorescence probes.

Dextran has been demonstrated to be a typical agent transported via the paracellular route, passing through tight junctions in a molecular weight‐dependent manner.^[^ ^53^ ^]^ Due to its small hydrodynamic radius, the penetration of dextran (10 kDa) into the surrounding hydrogels was observed (Figure 6b, left). Nevertheless, its diffusion was significantly suppressed by the endothelial barrier, particularly in the microchannel containing a bilayer structure (Figure 6c, left). BSA, a common serum protein with a molecular weight of 68 kDa, is typically restricted by the endothelial barrier under physiological conditions. In comparison to non‐endothelialized microchannels, minimal penetration of BSA was observed through the bilayer vascular structure after 2 h of incubation (Figure 6b, right). The time‐lapse diffusion of TRITC‐labeled BSA was monitored over 120 min by measuring fluorescence intensity through line‐scanning of the fluorescent images (Figure 6c, right). In naked channels, TRITC‐BSA rapidly diffused into the hydrogel, resulting in a high relative fluorescence intensity detected shortly after the injection of BSA solution. Similarly, in microchannels lining with a HUVEC monolayer, clear fluorescence signals of TRITC‐BSA were detected in the hydrogel, and the relative value increased with increasing incubation time, suggesting the limited barrier function of HUVEC monolayer in restricting BSA diffusion in this study. In contrast, BSA penetration was significantly suppressed in microchannels containing a bilayer structure.

This observation is consistent with previous studies showing that monolayers of cultured endothelial cells are usually 10–100 times more permeable to macromolecules than natural microvascular walls.^[^ ^54^ ^]^ Intercellular adherens and tight junction proteins are essential for regulating vascular endothelial permeability by preserving the integrity of the endothelial barrier.^[^ ^50^ ^]^ BMs proteins were reported to directly affect the barrier function of the vascular endothelium. For example, endothelial cell binding to laminin 511, but not 411, reduced permeability by enhancing the expression and localization of junctional proteins like VE‐cadherin at cell–cell borders.^[^ ^55^ ^]^ Meanwhile, the dense and interconnected structure of BM protein networks serves as a physical barrier, preventing the passage of cells and large molecules.^[^ ^51^ ^]^ SMCs and ECs interact through intercellular signaling, which enhances the secretion of BMs components and the maturation of BMs networks, thereby supporting the integrity and stability of the vascular endothelium. As shown in Figure 6d,e, rare penetration of TRITC‐BSA was detected around the bilayer microchannel even after 24 h incubation, with permeability reduced by 20‐fold compared to the blank hydrogel, closely resembling the barrier function of native vascular structures. It could provide more realistic and reliable in vitro data for drug screening, as well as for studying molecular transport across the endothelium and intracellular processes associated with changes in endothelial permeability. In addition, bioreactors have been integrated into a commercial cell‐culture plate (Figure S13c, Supporting Information), facilitating parallel experiments for toxicological and transport assays with ease.

## 3 Conclusion and Perspective

In conclusion, positively charged multilayer nanofilms, fabricated through LbL assembly of PLL and Dex, effectively recapitulated the barrier properties of BMs, successfully inhibiting the migration and sprouting of HUVEC. Compared to the previously reported A‐BMs composed of Col‐IV/LM NFs, the newly developed A‐BMs demonstrated markedly enhanced barrier properties. They effectively inhibited HUVEC migration over 14 d, matching the formation timeline of natural BMs in 3D tissues.^[^ ^30^ ^]^ Through systematic comparative analysis, it was determined that the improved barrier functions of the nanofilms could be attributed to their positive surface charge, which played a crucial role in inhibiting HUVEC migration or sprouting. This observation corresponds to previous reports that sprouting ECs possess a positively charged surface and typically migrate towards the cathode in a direct current electric field.^[^ ^31^, ^32^, ^33^ ^]^ Furthermore, the easy and biocompatible assembly process enables the creation of customizable shapes of A‐BMs for the on‐demand construction of organized tissue models, facilitating the understanding of integrated biological systems and ultimately aiding in the development of regenerative therapeutics. As an example, a 3D patterned vascular structure with clear lumen was engineered in a microchannel created in fibrin gel by a sacrificial template method. Positioned between SMCs layers and an ECs monolayer, PLL‐Dex NFs were in situ assembled following the topography of the microchannel to support the patterned cell co‐culture. The barrier function of the patterned bilayer, demonstrated using dextran and BSA, highlights the potential of this bioassay system for conducting in vitro drug permeability assays.

The successful implementation of this model will now be followed by efforts to optimize the manufacturing process to increase the yield and throughput of A‐BMs fabrication. Advanced techniques such as liquid handling robots, 3D printing, and microfluidics present promising approaches for high‐throughput LbL assembly.^[^ ^56^ ^]^ Integrating LbL assembly with these technologies could enhance the assembly efficiency, ensure the uniform coating thickness, and maintain consistent material properties, facilitating the transition from controlled laboratory settings to larger‐scale production for industrial or clinical uses. Moreover, the in situ construction and application of these new A‐BMs in more complex tissues, such as the irregular lamellar architecture in the liver and intestinal wall, holds great potential. In addition, the current PLL/Dex NFs effectively inhibited cell migration only in HUVEC. Further exploration of the barrier function of A‐BMs in blocking the infiltration of other cell types, such as mesenchymal cells, could support the development of anti‐fibrosis and anti‐adhesive products for applications like abdominal wall hernia repair.

## 4 Experimental Section

### PLL/Dex Multilayered Nanofilm Fabrication and Characterization

A 27 MHz quartz crystal microbalance (QCM, AFFINIX Q8, ULVAC SHOWCASE, Kanagawa, Japan) was used to quantitatively analyze the LbL assembly of PLL (Mw 15000–30000, 167‐12671, FUJIFILM Wako, Osaka, Japan) and dextran (Dex, Mw 60000, FUJIFILM Wako, Osaka, Japan) on a gold‐coated quartz crystal sensor using previously reported protocols.^[^ ^28^, ^57^, ^58^, ^59^ ^]^ First, QCM electrodes were cleaned with piranha solution (fresh mixture of H_2_SO_4_/40% H_2_O_2_ aqueous solution = 3:1 (v/v)) for 3 min, three times. Prior to use, QCM electrodes were rinsed with Milli Q and dried with N_2_. Temperature was maintained at 37 °C in all experiments. For the QCM study of film formation, PLL was coated as the first layer and Dex was deposited subsequently. For each deposition step, 100 µL of 50 × 10^−3^ m Tris‐HCl buffer solution (pH 7.4) was first added to each well. Under gentle stirring with an automatic stirring rod, 5 µL of polymer solution (2 wt% in 50 × 10^−3^ m Tris‐HCl buffer) was gently added to each well and the frequency was recorded in real‐time. Each adsorption durations was 15 min until the equilibrium. Between each step, the electrodes were washed three times with 1 × 10^−3^ m Tris‐HCl buffer solution (pH = 7.4) to remove excess and nonabsorbed polymers. The alternate steps were repeated until five bilayers, and the developed nanofilm was denoted as PLL/Dex NFs. The deposited amount of each polymer in each step was calculated referring to the Sauerbrey equation (1).^[^ ^35^, ^60^ ^]^ The assembly of each nanofilm was carried out in at least three independent channels. The frequency variations of the three sets of assembly were recorded, averaged, and then the average thickness of the nanofilm was calculated. Other multilayered films were also fabricated using the methods described above, including Col‐IV/LM NFs and PLL/DS NFs. The multilayered films were assembled on glass substrates for further characterization.

(1) $$-\Delta m\;\left(\mathrm{ng}/cm^{2}\right)=0.62\Delta F\left(\mathrm{Hz}\right)$$

### AFM Investigation

The prepared PLL/Dex NFs on a glass substrate were air‐dried, and the samples were subsequently imaged using AFM in tapping mode under ambient conditions. For imaging the topographical surface of the nanofilms, highly doped, gold‐coated silicon cantilevers (PPP‐NCSTAuD, NANOSENSORS, Neuchatel, Switzerland), with a resonant frequency of 160 kHz and a nominal spring constant of 7.4 N m^−1^, were used.

Thickness measurements were taken using an AFM tip‐scratch method.^[^ ^61^, ^62^, ^63^ ^]^ Briefly, a small square area (1.0 × 1.0 µm) was scanned in the contact mode to eliminate the deposits without causing damage to the glass substrate. Subsequently, a larger window (10 × 10 µm) was examined in tapping mode to ascertain the height differential, which corresponded to the actual thickness of the nanofilms. Notably, the depth of the scratches increased proportionally with the duration of the scratching process, until a point was reached where no further changes were observed.

### Evaluation of Stability and Protein Adsorption

The stability and protein adsorption of the multilayered nanofilms was analyzed using QCM. After the assembly of PLL/Dex NFs on QCM electrodes, the quantification of bovine serum albumin (BSA, 9048‐46‐8, Sigma‐Aldrich, St. Louis, MO) and fibronectin (FN, 289‐149‐2, Sigma‐Aldrich, St. Louis, MO) adsorption was performed using the QCM frequency shift, as detailed above. Similarly, the stability was assessed by immersing the assembled nanofilms in both PBS and DMEM medium supplemented with 10% FBS for over 1 h to monitor the frequency changes using QCM.

### Surface ζ Potential

Multilayered films were assembled on glass substrates (4 mm × 5 mm) following the previously mentioned assembly conditions. Their surface ζ potential was evaluated via phase analysis light scattering that was previously described by Corbett.^[^ ^64^, ^65^ ^]^ Briefly, glass substrates coated with the prepared nanofilms were stabilized on a holder positioned between electrodes and then submerged in a ZEN1020 plate cell (Surface zeta potential cell, Malvern Panalytical), containing a solution of narrowly dispersed tracer particles. The velocity of the tracer particles, affected by an alternating current field, was measured using phase analysis light scattering. The combined effects of particle electrophoretic migration and electro‐osmotic flow near the solid–liquid interface determined the overall velocity of the tracer particles at various positions. As the testing position shifts further from the sample surface, the impact of electro‐osmotic flow decreases, ultimately leading to a situation where the observed mobility is entirely due to electrophoretic migration. The values obtained were plotted against surface displacement, and the surface ζ potential was calculated by extrapolating the data to zero displacement. The contribution arising exclusively from the surface (ζ_surface_) is calculated from Equation (2). The surface ζ potential of films was evaluated using a Zetasizer nano ZS. 250 nm of Nile Red‐polystyrene nanoparticles (0.0005% w/v, FP‐0256‐2, Spherotech Inc., Lake Forest, USA) in 50 × 10^−3^ m Tris‐HCl buffer solution (pH 7.4, r.t.) were used as tracer particles. Mobility measurements were carried out at distances of 125, 250, 375, 500, and 1000 µm away from the sample surface. Three samples of each nanofilm were prepared for the measurement of surface ζ potential and then averaged.

(2) $$\zeta_{\mathrm{surface}}=-\mathrm{intercept}+\zeta_{\mathrm{particle}}$$

### Cell Culture

NHDFs (CC‐2509) (Passage: 5–8) were cultured with DMEM supplemented with 10% FBS and 1% penicillin/streptomycin, in an atmosphere containing 5% CO_2_ at 37 °C. HUVEC (C2517A) (Passage: 5–6) and GFP expressed HUVEC (GFP‐HUVEC, Angio‐Proteomie, Massachusetts, USA) (Passage: 5–6) were cultured in endothelial cell growth medium‐2 (EGM‐2, CC‐3162). AoSMC (CC‐2571) (Passage: 2–3) were cultured in smooth muscle cell growth medium‐2 (SmGM‐2, CC‐3182). For tissue culture, mixed culture media were used: DMEM/EGM‐2 (1:1, v:v) for the co‐culture of NHDF and HUVEC, SmGM‐2/EGM‐2 (1:1, v:v) for the co‐culture of AoSMC and HUVEC. All media and cells were purchased from Lonza, Basel, Switzerland.

### Evaluation of Cell Adhesion and Cell Functions on Multilayered Nanofilms

Various nanofilms were assembled on the membrane of 24‐well inserts. 1.0 × 10^5^ of NHDF and HUVEC were seeded, respectively, on the nanofilms, and a blank insert membrane served as the control. Both NHDF and HUVEC were cultured for 24 and 48 h, respectively, and the cytoskeleton was stained to examine the morphology of adhered cells. Furthermore, HUVECs were cultured for a period of 5 d to analyze the expression of CD31 and ZO‐1.

### Construction of a Patterned 3D Cell Co‐Culture Structure

PLL/Dex NFs were engineered to reside between fibroblast layers and endothelial cells, creating a “sandwich” configuration that mimics the architecture of natural tissue. Specifically, 250 µL of 1.0 × 10^6^ NHDF suspension was seeded into a 24‐well insert that had been pre‐incubated in a laminin (0.004 wt%)/Tris‐HCl solution (50 × 10^−3^ m, pH = 7.4) at 37 °C for 1 h to facilitate cell adhesion. 1 mL of DMEM was added to the underlayer of the transwell and the samples were then placed in an incubator at 37 °C for 24 h to allow NHDF attachment. After three washes with PBS, 250 µL of PLL and Dex (0.1 wt%)/Tris‐HCl (50 × 10^−3^ m, pH 7.4) solutions were sequentially added to the insert, with each addition followed by an incubation at 37 °C for 15 min. 1 mL of Tris‐HCl buffer solution was maintained beneath the inserts. Following each step, the samples were rinsed once with a 1 × 10^−3^ m Tris‐HCl buffer solution to eliminate excess and non‐adsorbed polymers. This alternating adsorption process was repeated four times, leading to the formation of PLL/Dex NFs. A volume of 250 µL of 1.0 × 10^5^ HUVEC suspension in mixed medium (DMEM/EGM‐2 at a 1:1 ratio) was placed on the assembled nanofilms, and 1 mL of the mixed medium was added into the underlayer of the transwell. The samples were then incubated for 24 h to facilitate HUVEC adhesion, after which the medium was replaced with 2 mL of mixed medium per well. The tissues were cultured for 14 days, with the medium being replaced once every two days. A patterned cell co‐culture structure, lacking nanofilms, served as the control sample. The efficacy of cell separation between NHDF and HUVEC using PLL/Dex nanofilms was evaluated and compared to other films. To determine the precise locations of both NHDF and HUVEC, immunofluorescence staining and histological staining techniques were employed. The layered co‐culture structure of AoSMC and HUVEC was also prepared in the same manner.

To verify the integrity of this patterned structure, FITC‐labeled PLL (Mw 15000–30000, P3543‐10MG, Sigma‐Aldrich, St. Louis, MO) and TRITC‐Dex (Mw 500000, 52194‐1G, Sigma‐Aldrich, St. Louis, MO) were incorporated between the layers of NHDF and HUVEC. In conjunction with immunofluorescence staining of NHDF and HUVEC, the fluorescent PLL/Dex NFs were visualized using a CLSM (FV3000, Olympus, Japan).

### Fabrication of a Tubular Vascular Structure in 3D Hydrogel

A vascular‐like structure was constructed in 3D fibrin gel as previously reported with modifications.^[^ ^47^ ^]^ Glass coverslips (Ø 15 mm, C015001, Matsunami, Osaka, Japan) were pasted to the bottom of a bioreactor using non‐cytotoxic medical grade double‐sided tape (ARcare 90106NB, Adhesives Research, Glen Rock, USA). A templating scaffold of P*cyclo*PrOx with a diameter of 350 µm was placed on the saddle supports of the bioreactors and then fixed with P*cyclo*PrOx solution (40 wt%). Following adhesive drying at ambient temperature, bioreactors with P*cyclo*PrOx scaffolds were sterilized under UV light for 10 min. Then, 400 µL of fibrin gel was used to fill the main chamber of the bioreactor and the scaffolds were embedded in the fibrin gel. Fibrin gel was prepared by mixing 266.7 µL of 5 wt% fibrinogen solution (F8630‐5G, Sigma‐Aldrich, St. Louis, MO) and 133.3 µL of 25 U mL^−1^ thrombin solution (E6758‐500G, Sigma‐Aldrich, St. Louis, MO) at room temperature and then incubated at 37 °C for complete gelation. Once the hydrogel formed, cold PBS solution was added to the bioreactor chambers to dissolve the P*cyclo*PrOx scaffold, leaving empty microchannels.

10 µL of 3.0 × 10^5^ AoSMC suspension was gently injected into the microchannel that was pre‐incubated in fibronectin solution (0.01 wt%) at 37 °C for 1 h to facilitate cell adhesion. Following cell seeding, the bioreactor was transferred to an incubator. After 3 h incubation, the bioreactor was inverted and incubated for another 3 h to ensure the homogeneity of the cell attachment. 1.0 mL of SmGM‐2 was added to each side chamber and 0.5 mL SmGM‐2 was added in the main chamber. Cell attachment was confirmed by cytoskeleton staining of AoSMC after 2 d culture. HUVEC was also seeded following the same protocol, but the incubation time after injecting the cell suspension was 15 min for each side. A confluent HUVEC monolayer attached in the microchannel was confirmed by CD31 staining after 5 days culture. A tubular multicellular structure was fabricated by successively seeding AoSMC and HUVEC. Briefly, AoSMC was seeded and cultured for 2 d, and then in situ LbL assembly of PLL/Dex NFs was performed on the AoSMC surface before HUVEC seeding. Samples were placed on a rocker platform set to a continuous perfusion mode with an inclination of ≈30°. The mixed medium (SmGM‐2/EGM‐2 at 1:1 ratio) was then used for tissue culture. The medium was changed every day.

### Permeability Assay of 3D Patterned Vascular Structure

To quantify and visualize endothelial barrier function, TRITC‐conjugated dextran (10 kDa, D1817, Invitrogen, California, USA) and BSA (A2289‐50MG, Sigma‐Aldrich, St. Louis, MO) solutions were prepared at a concentration of 0.5 mg mL^−1^ in cell culture medium.^[^ ^47^, ^66^ ^]^ Each solution was introduced into the inlet reservoir of their respective microchannels for analysis. The permeability assay was assessed by analyzing the diffusion of molecules labeled with TRITC probes into the fibrin gel matrix under three conditions: bare microchannel (blank), microchannel lined with a HUVEC monolayer, and microchannel containing a bilayer composed of AoSMC and HUVEC within hydrogels. Confocal fluorescence images were captured at 0 h (immediately after injection) and subsequently every 15 min for a total of 2 h. The diffusion of BSA was further assessed after 24 h of culture. Fluorescence intensity was measured by line‐scanning over a distance of 500 µm, with 0 µm representing the location of hydrogel surface, using image J software. The relative fluorescence intensity at a distance of 50 µm from the hydrogel interface, compared to the value at the center of the microchannels, was used to indicate the relative amount of penetrated molecules.

### Formation of Natural BMs

To assess the secretion of Col‐IV by cells co‐cultured with PLL/Dex NFs, immunofluorescence staining for Col‐IV was conducted and subsequently analyzed using the FV3000 microscope. A control sample consisting of a patterned cell co‐culture without nanofilms was used for comparison. A laminin coating (0.004 wt% in 50 × 10^−3^ m Tris‐HCl) was applied between NHDF and HUVEC layers instead of Col‐IV/LM NFs to compare distribution of naturally deposited Col‐IV with those cultured with PLL/Dex NFs. The patterned tissues were cultured for 7 d, followed by staining with anti‐Col‐IV and anti‐CD31 antibodies, and the morphology of Col‐IV networks was visualized using an FV3000 microscope.

### Cell Tracker Staining

CellTracker Deep Red (C34565, Invitrogen, California, USA) was initially prepared in dimethyl sulfoxide (DMSO) at 10 × 10^−3^ m and subsequently diluted to a 1:1000 volume ratio in FBS‐free medium. Pre‐cultured cells underwent three PBS washes before a 40‐min incubation with the dye at 37 °C. Following aspiration of the dye‐containing medium and three additional PBS washes, the cells were incubated in media at 37 °C for a minimum of 24 h prior to detachment.

### Immunofluorescence Staining

The 2D monolayer or 3D tissues cultured in a 24‐well insert were rinsed three times with PBS and fixed by 4% paraformaldehyde (PFA, Fujifilm Wako, Osaka, Japan) for 15 min at room temperature. After three rinses with PBS, permeabilization was then carried out using 0.2% Triton‐X 100 (Sigma‐Aldrich, St. Louis, MO) for 30 min at room temperature. After PBS rinsing, 1 wt% BSA/PBS solution was added at room temperature for 30 min to block the unspecific staining of the antibody. The samples were then incubated with primary antibodies overnight at 4 ​°C: TRITC‐phallotoxin (084K0443, Sigma‐Aldrich, St. Louis, MO), anti‐CD31 antibody (mouse anti‐human, NCL‐CD31‐1A10, Leica, Wetzlar, Germany), and anti‐rabbit, ab28364, Abcam, Cambridge, UK) were diluted in 1% BSA in PBS at 1/100, while anti‐ZO‐1 (mouse anti‐human, ZO1‐1A12, Invitrogen, Waltham, MA) and anti‐collagen‐IV (monoclonal mouse anti‐human, clone CIV 22, Thermo Fisher Scientific, Waltham, MA) were diluted in 1% BSA in PBS at 1/50. After rinsing with PBS three times, samples were then incubated for 2 ​h at room temperature in the dark with secondary antibodies diluted at 1/200 in 1% BSA in PBS: goat anti‐mouse, Alexa Fluor 488 (A11001), Alexa Fluor 546 (A11003), Alexa Fluor 647 (A21235) (Thermo Fisher Scientific, Waltham, MA), and goat anti‐rabbit, Alexa Fluor 488 (ab150077, Abcam, Cambridge, UK). The nuclei were stained with Hoechst 33342 (H3570, Thermo Fisher Scientific, Waltham, MA) that was diluted 1000 times in PBS. After staining, the samples were washed with PBS three times. Fluorescence images were then observed with an FV3000. Images were digitized using Imaris software (ver. 9.2.1, Oxford Instruments, Bitplane, Belfast, UK). Adhered cell number, coverage area, expression of CD31, ZO‐1, and Col‐iv, migrated cell number, and depth were quantified using ImageJ.

### Histology Staining

Insert cultured tissues were rinsed with PBS three times and fixed in 4% PFA for 15 min at room temperature. Another three washes with PBS were performed, and the tissues were then sent to the Applied Research Company for paraffin embedding. Sectional samples were stained with hematoxylin and eosin (H&E) and CD31. Brightfield images were captured using an FL Evos Auto microscope (Thermo Fisher Scientific, MA, USA).

### Cell Viability

*Trypan Blue Stain Kit*: After the LbL assembly process performed on NHDF layers in situ, the tissues were incubated for 24 h. On the second day, the samples were rinsed with PBS and subsequently incubated in 200 µL of 0.25% trypsin (209‐19182, Fujifilm Wako, Osaka, Japan) with 0.02% EDTA (E6758‐500G, Sigma Aldrich, St. Louis, MO) at 37 °C for 20 min. During this process, the digested tissues were gently pipetted twice for the cell isolation. An equivalent amount of medium was added to stop the digestion process. Isolated cells were stained with 0.4% trypan blue (2420718, Thermo Fisher Scientific, Waltham, MA) to quantify the cell number. Live and dead cell number was counted by an automated cell counter (Countess II, Thermo Fisher, Waltham, MA).

*LIVE/DEAD Viability Assay*: With the same trypan blue stain kit, tissues cultured on the next day were rinsed with PBS three times and incubated in 250 µL of PBS solution containing Calcein AM and EthD‐1/PBS (2 µmol L^−1^) at 37 °C for 45 min in the dark. Cell conditions were imaged at the surface, middle and bottom of the 3D tissue using an FV3000.

### Statistical Analysis

In this study, all values are presented as means ​± ​standard deviation (SD). Statistical analysis of the data was performed with Student's *t*‐test when more than two samples were compared. Error bars represent standard deviations. Test gave *p*‐values considered significant if *p < 0.05.

## Conflict of Interest

The authors declare no conflict of interest.

## Supporting information



Supporting Information

Supporting Information Video S1

Supporting Information Video S2

## Data Availability

The data that support the findings of this study are available from the corresponding author upon reasonable request.
