# Supplementary material for: Outermost Cationic Surface Charge of Layer‐by‐Layer Films Prevents Endothelial Cells Migration for Cell Compartmentalization in Three‐Dimensional Tissues
Source: Adv Sci (Weinh). 2025 Feb 22;12(19):2417538. doi: 10.1002/advs.202417538 (PMC12097075; doi:10.1002/advs.202417538)
Supplement: Supplementary file 1 — Supporting Information [file ADVS-12-2417538-s003.docx]

**Outermost Cationic Surface Charge of Layer-by-Layer Films Prevents Endothelial Cells Migration for Cell Compartmentalization in Three-Dimensional Tissues**

*Jinfeng Zeng,^1,2^ Sven* *Heilig^3^ Matthias Ryma,^3^ Jürgen Groll,^3^ Congju Li^1^ and Michiya Matsusaki^2,4^**

J. Zeng, C. Li

^1^College of Textiles, Donghua University, Shanghai 201620, China.

J. Zeng, M. Matsusaki

^2^Department of Applied Chemistry, Graduate School of Engineering, Osaka University, 2-1 Yamadaoka, Suita, Osaka 565-0871, Japan.

*Corresponding author, Email: [m-matsus@chem.eng.osaka-u.ac.jp](mailto:m-matsus@chem.eng.osaka-u.ac.jp)

*S. Heilig, M. Ryma, J. Groll*

^3^University of Würzburg, Pleicherwall 2, 97070 Würzburg, Germany.

M. Matsusaki

^4^Joint Research Laboratory (TOPPAN) for Advanced Cell Regulatory Chemistry, Osaka University, Suita, Osaka, Japan.


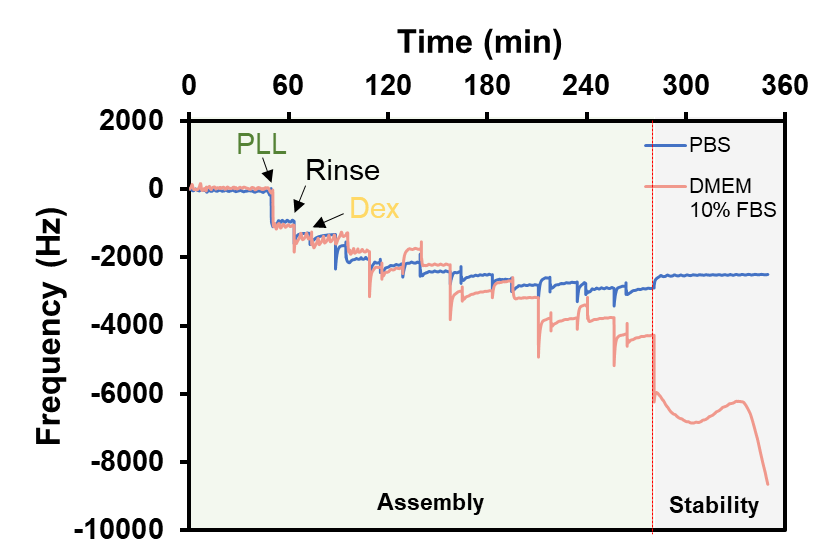


**Figure S1**. left: Frequency changes recorded by QCM for (PLL/Dex)_5_ as a function of time in 50 mM Tris-HCl buffer solution (pH=7.4, 37 °C). right: Frequency changes of assembled PLL/Dex NFs soaked in PBS and DMEM (10% FBS) for 60 min at 37 ℃.


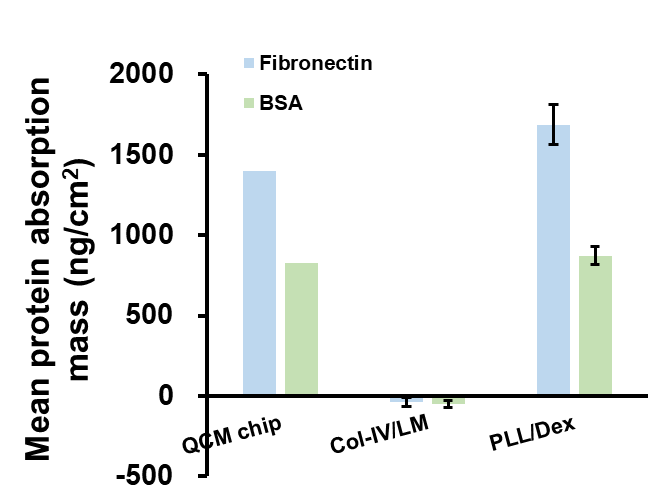


**Figure S2.** Protein adsorption on a bare QCM chip and LbL films is assessed through a 60 min-incubation using QCM. The concentrations of fibronectin and BSA are 0.02 wt% and 0.1 wt%, respectively.


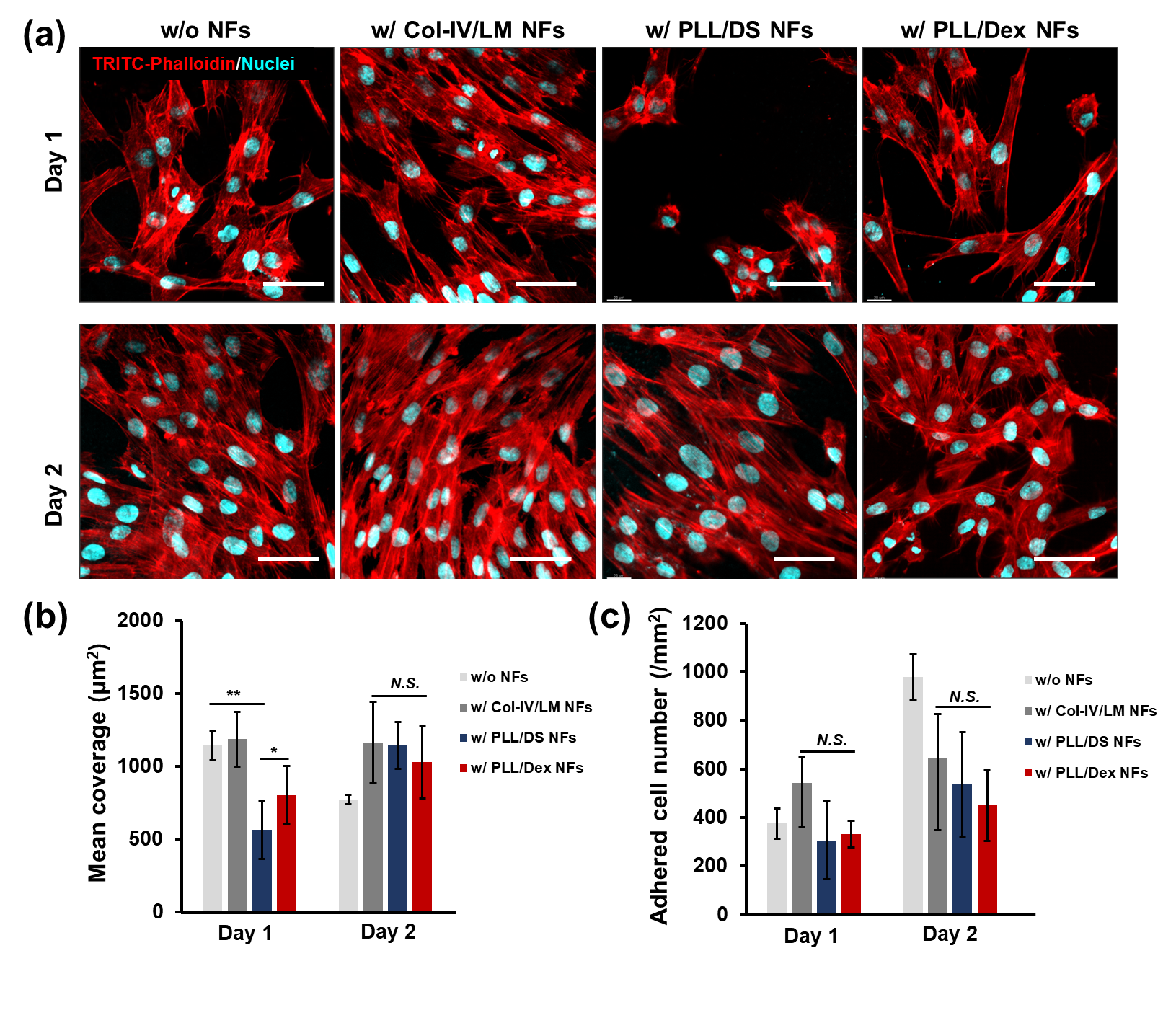


**Figure S3**. (a) Morphology of NHDF cultured on Col-IV/LM, PLL/DS and PLL/Dex NFs. A naked 24-well insert acts as the bare substrate for cell culture (w/o NFs). Cells are cultured for 2 days, after which the cytoskeleton is stained for actin visualization. Scale bar: 50 μm. Comparison of the (b) adhesion area of single cell and (c) adhered cell number on different substrates. n=3. **p*<0.05, ***p*<0.01. *N.S.* no significant difference.


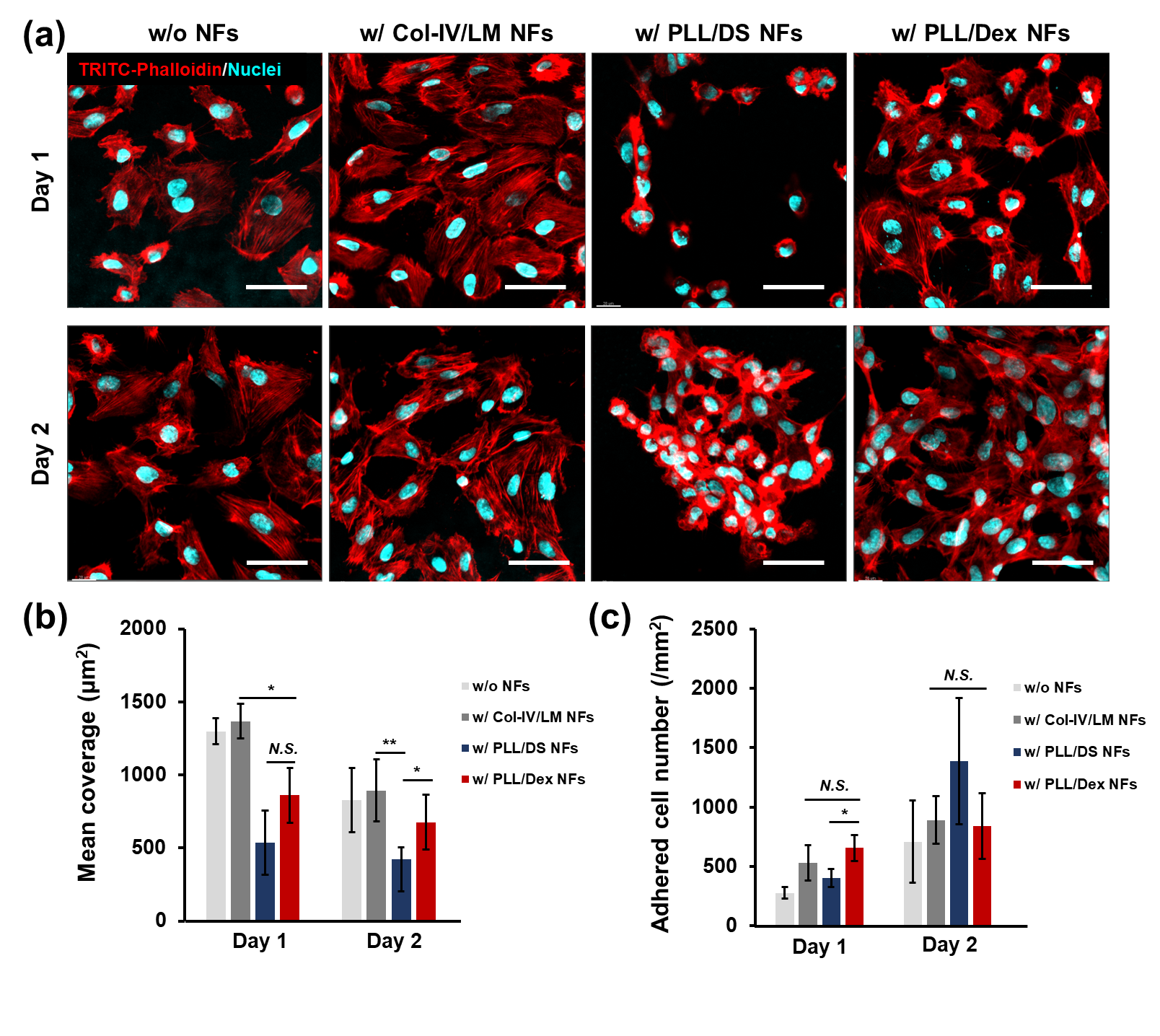


**Figure S4**. (a) Morphology of HUVEC cultured on Col-IV/LM, PLL/DS and PLL/Dex NFs. A naked 24-well insert acts as the bare substrate for cell culture (w/o NFs). Cells are cultured for 2 days, after which the cytoskeleton is stained for actin visualization. Scale bar: 50 μm. Comparison of the (b) adhesion area of single cell and (c) adhered cell number on different substrates. n=3. **p*<0.05, ***p*<0.01. *N.S.* no significant difference.


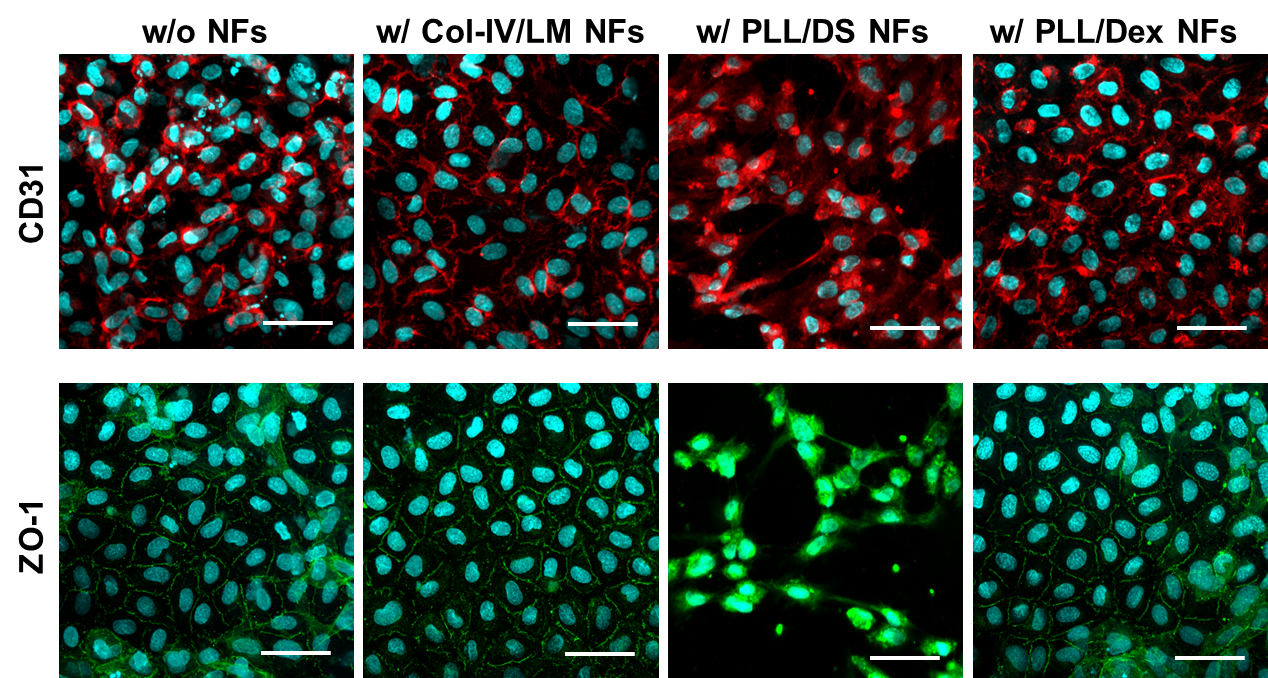


**Figure S5**. Immunofluorescence staining of HUVEC with CD31 and ZO-1 cultured on Col-IV/LM, PLL/DS and PLL/Dex NFs for 7 days, respectively. HUVEC monolayer cultured on a naked 24-well insert serves as the control sample (w/o NFs). Nuclei are stained with Hoechst 33342 shown in cyan. Scale bar: 50 μm.


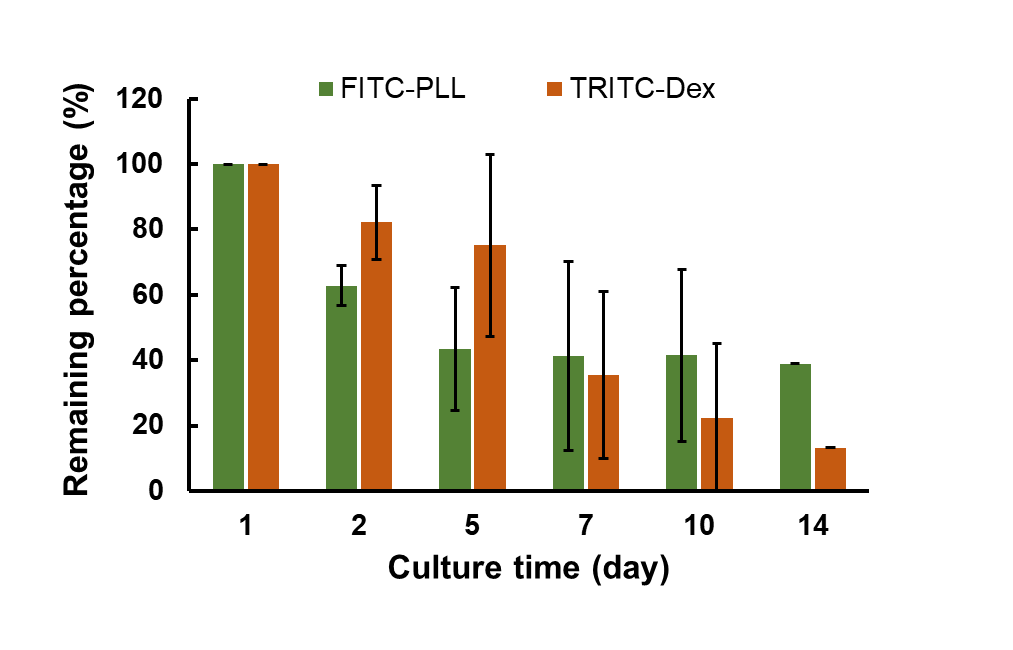


**Figure S6.** The remaining percentage of PLL and Dex during the co-culture with cells for 14 days.


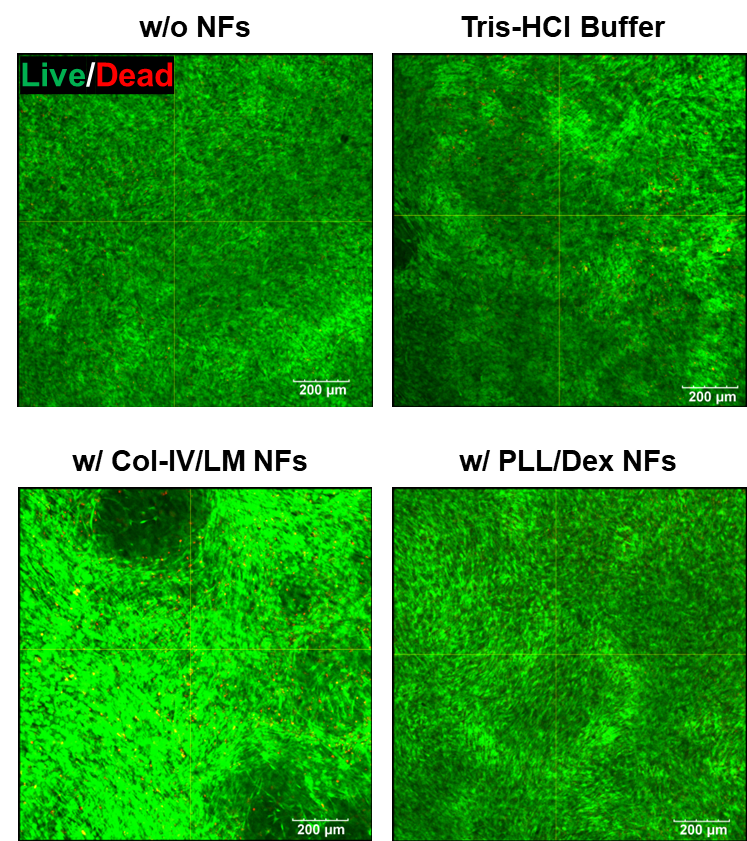


**Figure S7.** Cell viability of NHDF layers treated with *in-situ* LbL assembly. The Live/Dead staining was performed after the assembly and subsequent cultured overnight. Scale bar: 200 μm.


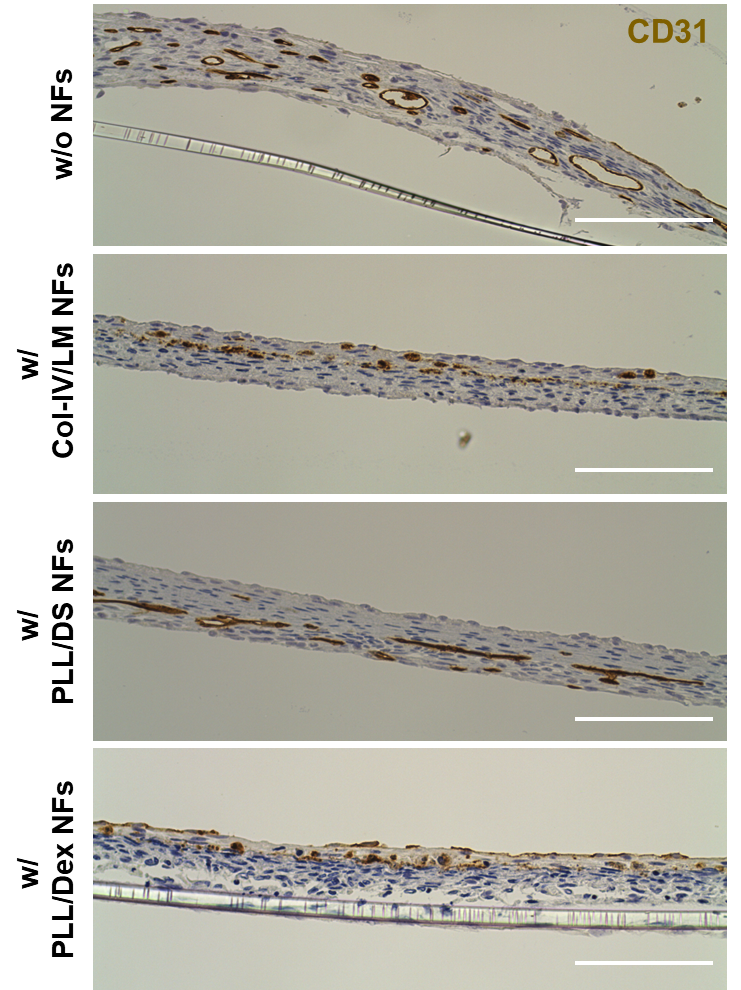


**Figure S8**. Histological observation of a cross-sectioned co-culture structure (HUVEC are stained with CD31, toluidine staining for nuclei). Scale bar: 100 μm.


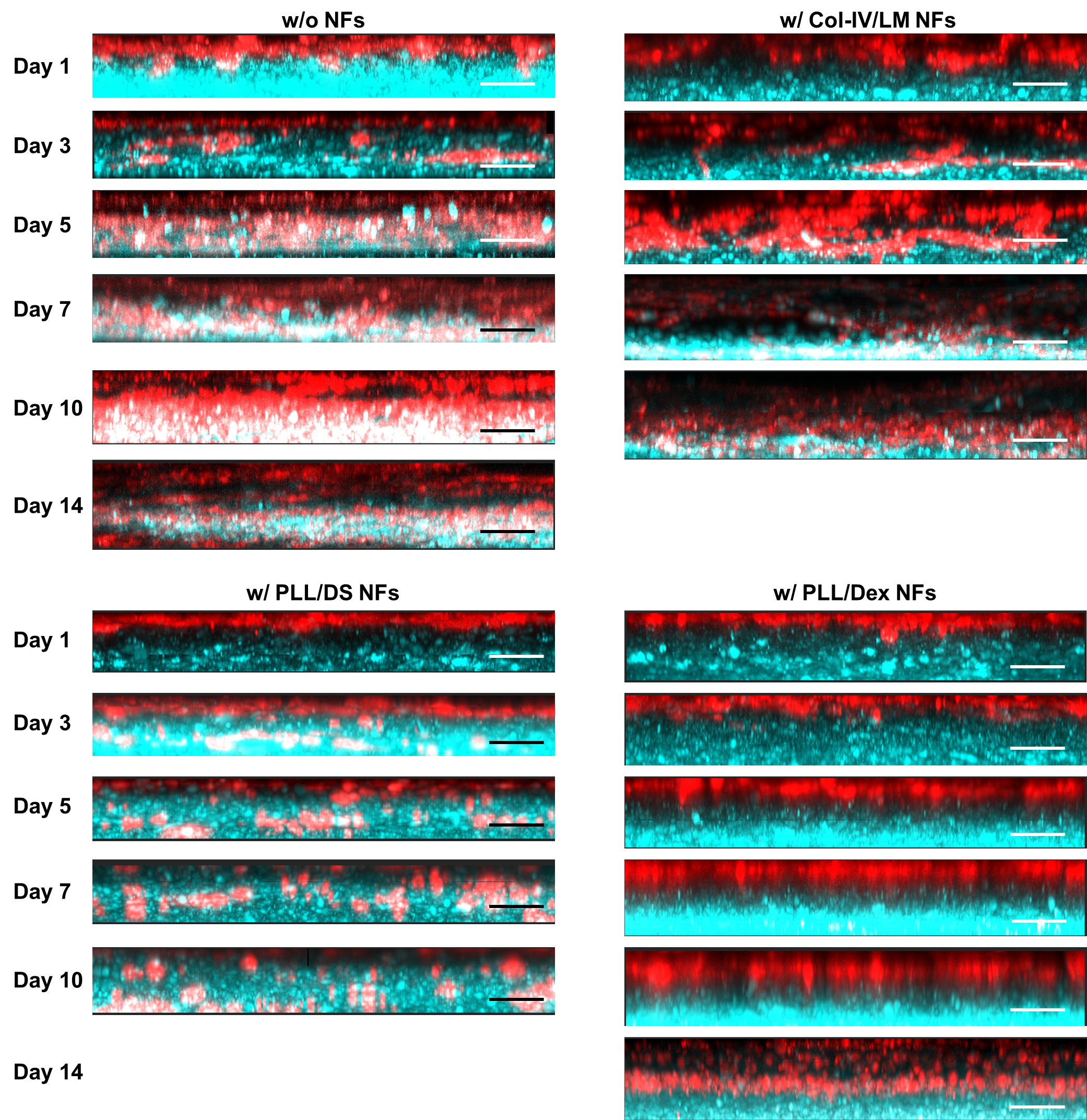


**Figure S9**. Cross-sectional CLSM images show the relative position of NHDF and HUVEC during 2 weeks of culture with various LbL NFs. Scale bar: 50 μm.


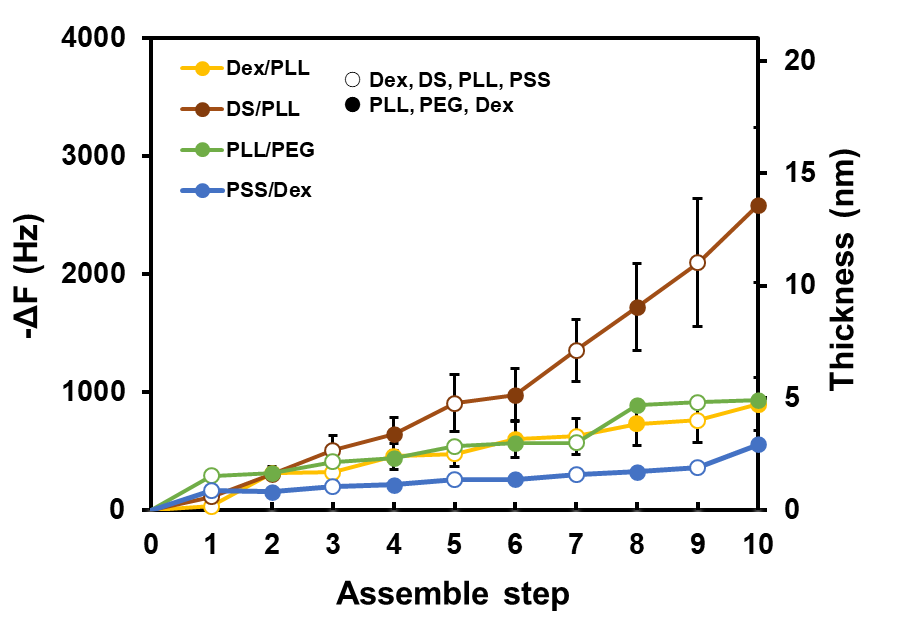


**Figure S10**. Frequency shifts and film thickness increases during assembly are summarized for various LbL films composed of various polymers. Frequency changes were recorded by QCM during assembly in 50 mM Tris-HCl buffer solution (pH=7.4, 37 °C). The concentration of each polymer is 0.1wt%.


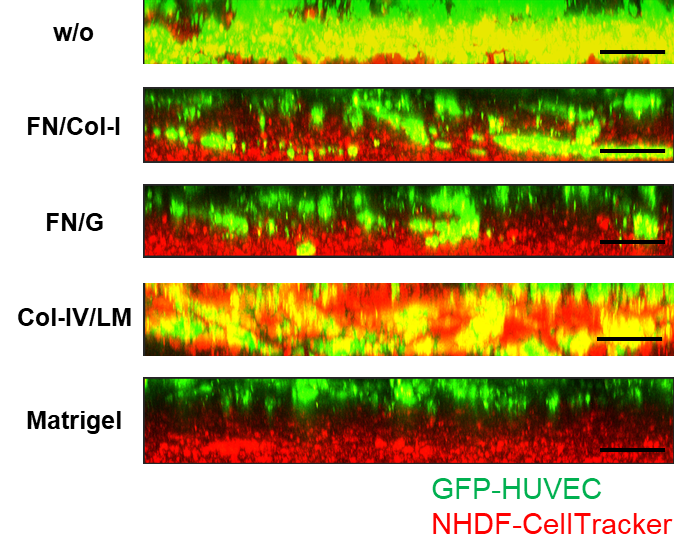


**Figure S11**. Cross-sectional CLSM images of co-culture system with different ECM protein nanofilms, which are observed after 7 days of culture. Scale bar: 50 μm.


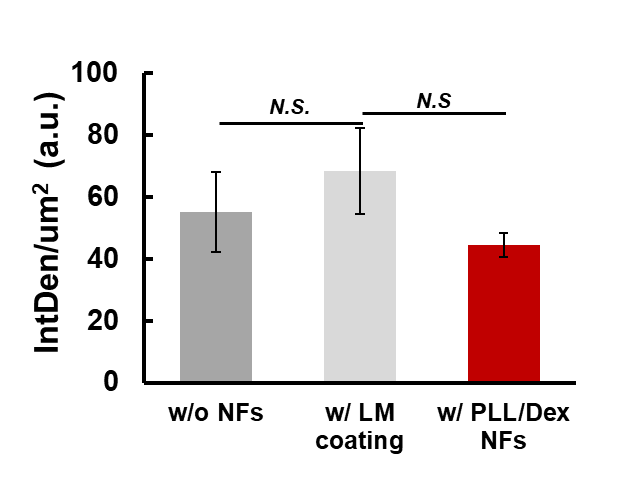


**Figure S12.** Quantified fluorescence intensity of Col-IV deposited in the co-culture systems. n=2.


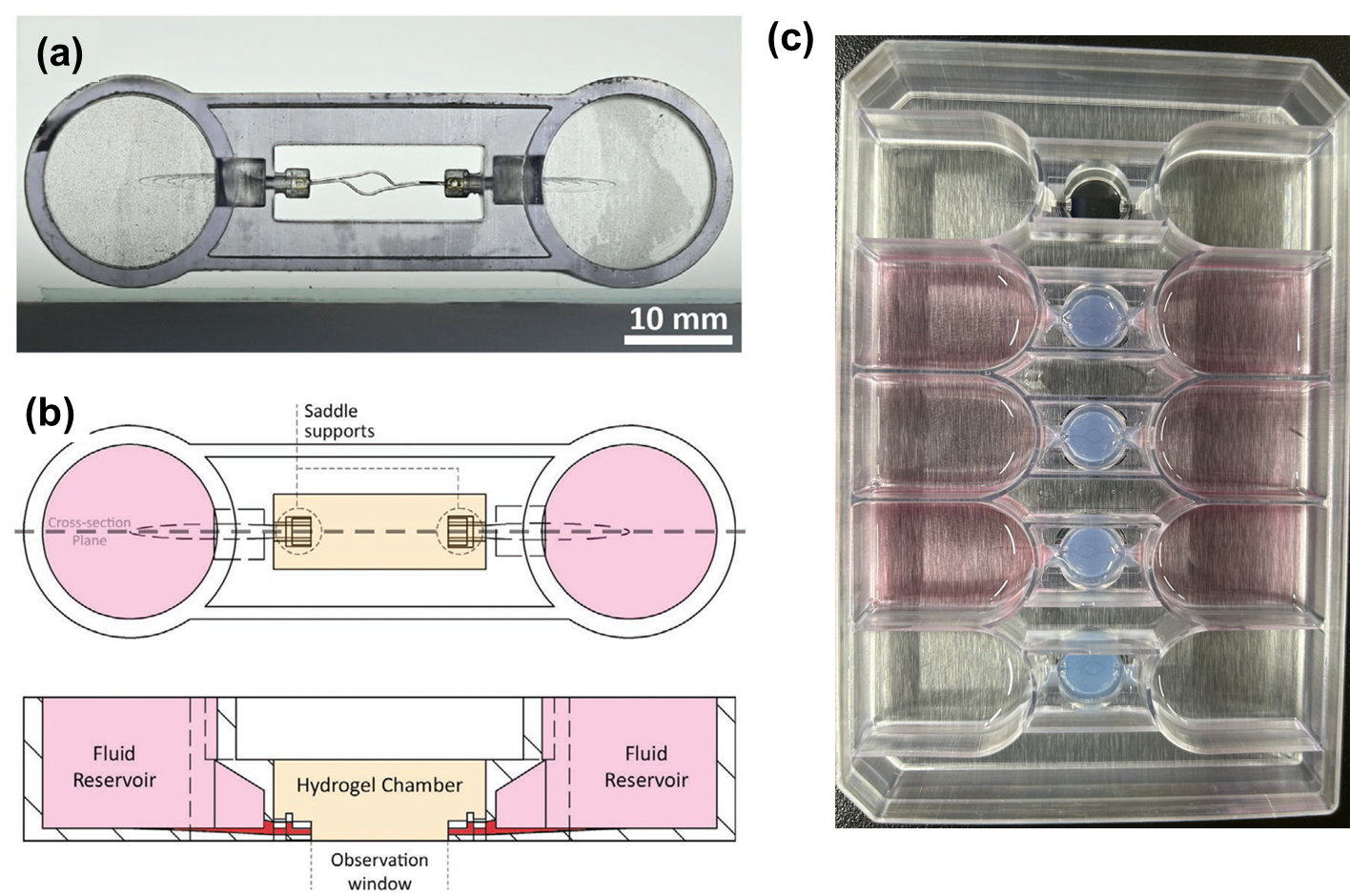


**Figure S13**. (a) Top-view photo of a bioreactor with PcycloPrOx scaffold embedded on the supporting saddles. (b) Design aspects of the bioreactor showing the different components (top) and a cross-section of the top view (bottom). Reproduced with permission from^[1]^ Copyright 2022 WILEY-VCH. (c) Digital photo of bioreactors constructed with blood vessel analogs in commercial cell culture plate.


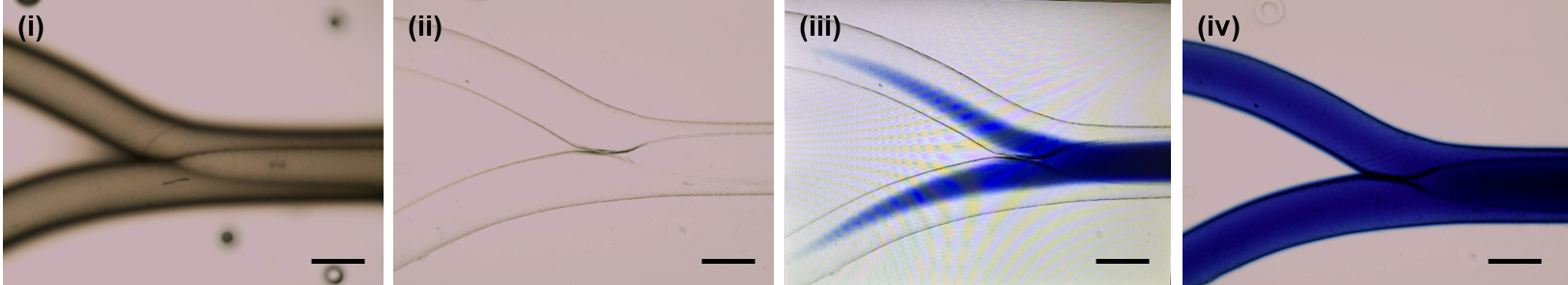


**Figure S14**. (i-ii) Phase contrast images of the embedding, dissolution of PcycloPrOx scaffold in fibrin gel under LCST. (iii-iv) The diffusion of microchannel using trypan blue. Scale bar: 500 μm.


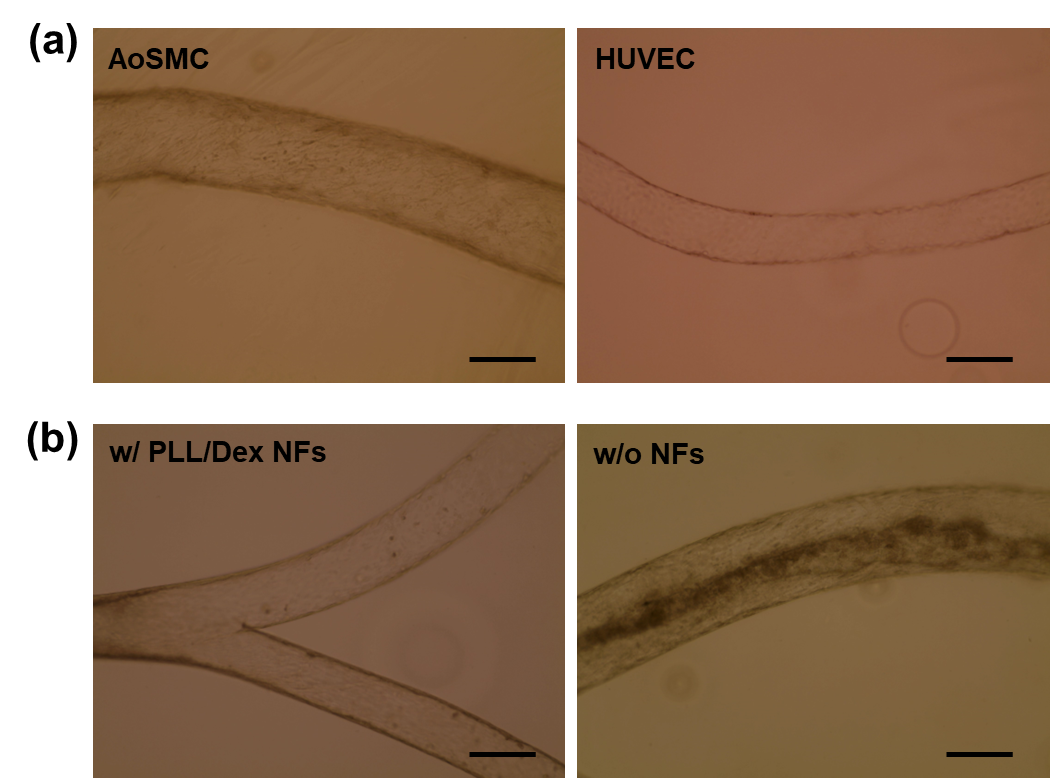


**Figure S15**. (a) Phase contrast images of attached AoSMC and HUVEC in the microchannels. (b) 3D co-culture of AoSMC and HUVEC in microchannels with (w/) or without (w/o) PLL/Dex NFs for 5 days. Scale bar: 500 μm.


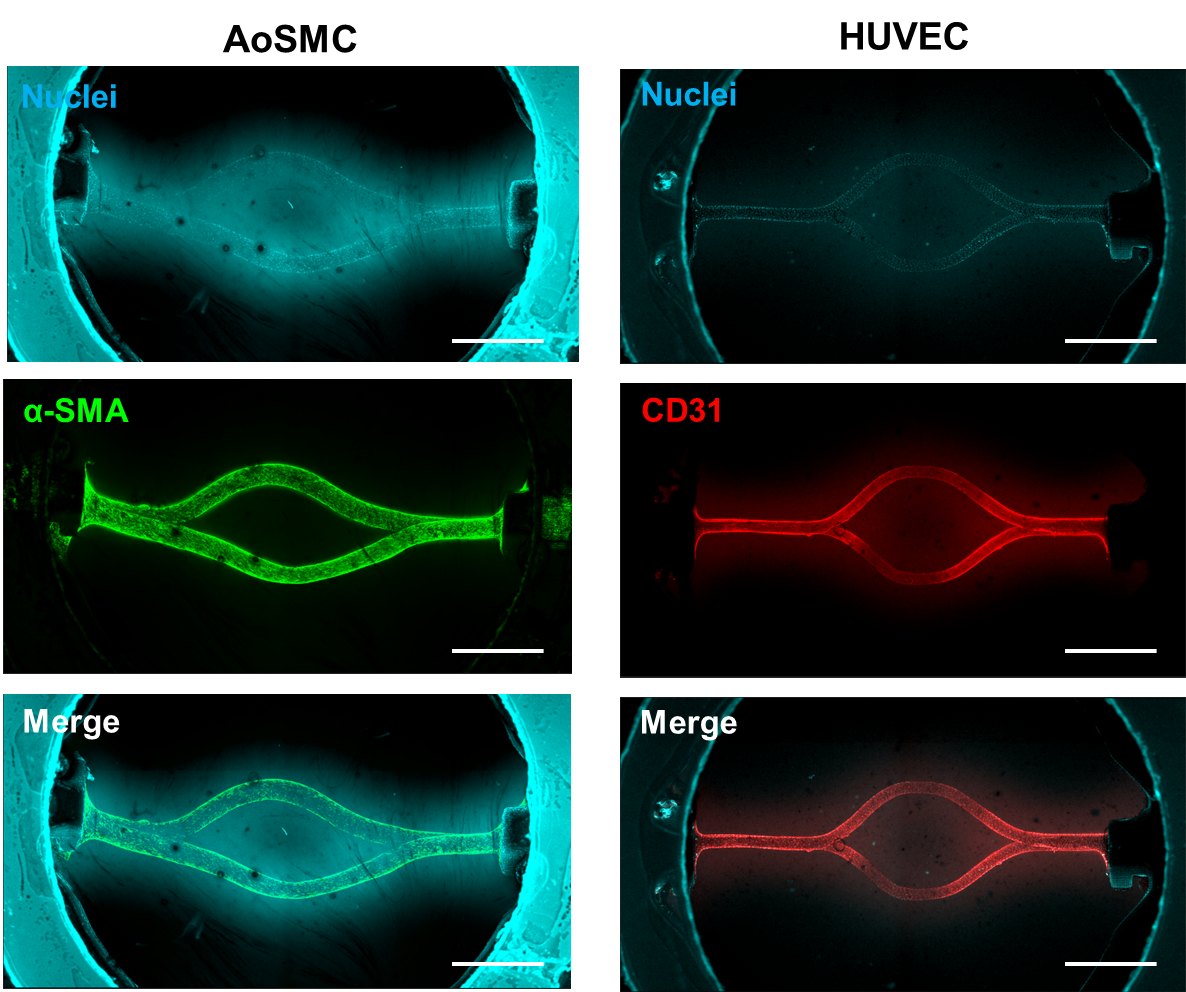


**Figure S16**. Large view of immunofluorescent staining of α-SMA for AoSMC and CD31 for HUVEC to show the attachment of two types of cells in microchannels. Nuclei are stained with Hoechst 33342 in cyan. Scale bar: 2000 μm.


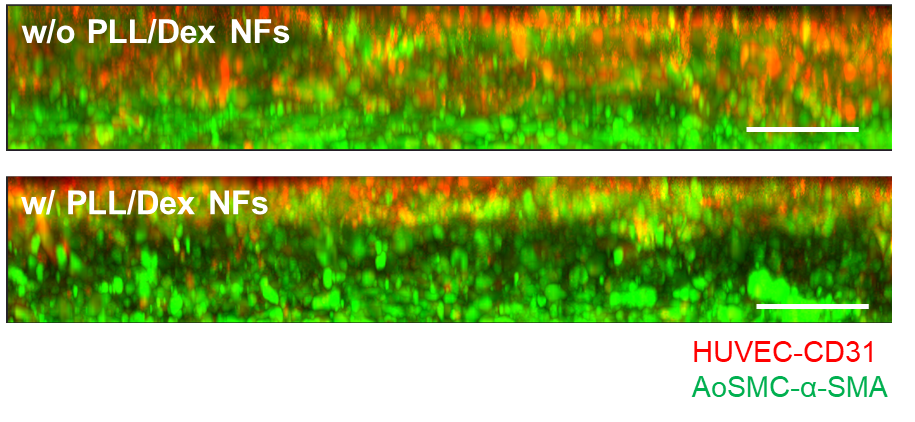


**Figure S17.** Cross-sectional CLSM images of co-culture system of HUVEC and AoSMC in 24-well insert with or without PLL/Dex NFs, which are observed after 5 days of culture. Scale bar: 50 μm.

**Reference**

[1] M. Ryma, H. Genç, A. Nadernezhad, I. Paulus, D. Schneidereit, O. Friedrich, K. Andelovic, S. Lyer, C. Alexiou, I. Cicha, J. Groll, *Advanced Materials* **2022**, *34*, 2200653.
